# A framework of NGO inside and outside strategies in the commercial determinants of health: findings from a narrative review

**DOI:** 10.1186/s12992-023-00978-x

**Published:** 2023-10-10

**Authors:** Belinda Townsend, Timothy D. Johnson, Rob Ralston, Katherine Cullerton, Jane Martin, Jeff Collin, Fran Baum, Liz Arnanz, Rodney Holmes, Sharon Friel

**Affiliations:** 1 Australian Research Centre for Health Equity, School of Regulation and Global Governance, Australian National University, Canberra, Australia; 2 Global Health Policy Unit, Social Policy, School of Social and Political Science, University of Edinburgh, Edinburgh, UK; 3 School of Public Health, University of Queensland, Herston, Australia; 4 Obesity Policy Coalition, Melbourne, Australia; 5 Stretton Health Equity & School of Social Science, University of Adelaide, Adelaide, Australia; 6 NCD Alliance, Geneva, Switzerland; 7 Foundation for Alcohol Research and Education, Canberra, Australia

**Keywords:** Commercial determinants of health, NGO, Civil society, Advocacy, Policy, Public health, Health policy

## Abstract

**Background:**

Public health scholarship has uncovered a wide range of strategies used by industry actors to promote their products and influence government regulation. Less is known about the strategies used by non-government organisations to attempt to influence commercial practices. This narrative review applies a political science typology to identify a suite of ‘inside’ and ‘outside’ strategies used by NGOs to attempt to influence the commercial determinants of health.

**Methods:**

We conducted a systematic search in Web of Science, ProQuest and Scopus. Articles were eligible for inclusion if they comprised an empirical study, explicitly sought to examine ‘NGOs’, were in English, and identified at least one NGO strategy aimed at commercial and/or government policy and practice.

**Results:**

One hundred forty-four studies met the inclusion criteria. Eight industry sectors were identified: extractive, tobacco, food, alcohol, pharmaceuticals, weapons, textiles and asbestos, and a small number of general studies. We identified 18 types of NGO strategies, categorised according to the target (i.e. commercial actor or government actor) and type of interaction with the target (i.e. inside or outside). Of these, five NGO ‘inside’ strategies targeted commercial actors directly: 1) participation in partnerships and multistakeholder initiatives; 2) private meetings and roundtables; 3) engaging with company AGMs and shareholders; 4) collaborations other than partnerships; and 5) litigation. ‘Outside’ strategies targeting commercial actors through the mobilisation of public opinion included 1) monitoring and reporting; 2) protests at industry sites; 3) boycotts; 4) directly engaging the public; and 5) creative use of alternative spaces. Four NGO ‘inside’ strategies directly targeting government actors included: 1) lobbying; 2) drafting legislation, policies and standards; 3) providing technical support and training; and 4) litigation. NGO ‘outside’ strategies targeting government included 1) protests and public campaigns; 2) monitoring and reporting; 3) forum shifting; and 4) proposing and initiating alternative solutions. We identified three types of NGO impact: substantive, procedural, and normative.

**Conclusion:**

The analysis presents a matrix of NGO strategies used to target commercial and government actors across a range of industry sectors. This framework can be used to guide examination of which NGO strategies are effective and appropriate, and which conditions enable NGO influence.

**Supplementary Information:**

The online version contains supplementary material available at 10.1186/s12992-023-00978-x.

## Introduction

Public health scholarship focused on the commercial determinants of health has uncovered a wide range of strategies used by commercial actors to promote sales of their products and influence government regulation [1, 2]. Documented strategies include lobbying and political donations, partnering with governments, engaging in multistakeholder platforms, funding research biased in favour of industry, co-opting health professionals and policymakers to promote industry objectives, intimidating critics, undermining legitimate science and reframing debate, and promoting corporate social responsibility initiatives [3–8]. The recent Lancet series on the Commercial Determinants of Health (CDoH) defines the commercial determinants as the ‘systems, practices and pathways through which commercial actors drive health and equity’ [9].

Calls for a ‘public health playbook’ point to the need for public health actors to develop a suite of strategies to counter industry power and influence [7]. These actors include experts, think tanks and non-government organisations (NGOs) who can play an important role in the policy process. NGOs are one particularly important but understudied group of actors who comprise much of civil society and play important roles in holding government and industry actors to account. Within the literature on CDoH, three recent studies emphasise the role of NGOs in agenda setting and acting as ‘watchdogs’ monitoring and reporting on government and commercial practices [10–12]. Other studies have highlighted the lobbying role of NGOs targeting government and intergovernmental actors [2, 13]. The range of strategies that NGOs use, however, is not well documented, particularly strategies directly targeting commercial practices. NGO tactics tend to be reported in case studies or issues area (e.g. tobacco), and not across domains. There is significant scope for learning lessons from what NGO strategies have been used across CDoH domains, which this review intends to identify. Furthermore, NGO influence is poorly conceptualised in the health governance literature [10]. This review aims to address these knowledge gaps by first mapping known NGO strategies used in the CDoH, and second, unpacking how NGO impact is conceptualised within this literature.

### Analytical framework

To conceptualise the range of strategies used by NGOs in the commercial determinants of health, we draw on a political science typology of ‘inside’ and ‘outside’ strategies developed in policy studies by Colli and Adriaensen [14]. This typology provides a useful heuristic for identifying the suite of strategies NGOs use to target different actors through political (government) and economic (market) spheres of action. In addition to clarifying the different targets of NGO activity (i.e. commercial or government), this typology pays attention to ‘inside’ and ‘outside’ strategies. ‘Inside’ strategies involve direct contact with the target actor, such as holding private meetings or responding to government consultations [15]. In contrast, ‘outside’ strategies aim to generate public attention to the target and increase public and political salience of the issue, such as through public campaigns or public protests [16]. ‘Inside’ strategies targeting commercial actors, for example, include cooperating with companies and shareholder activism, while outside strategies include protests, media campaigns and boycotts. In contrast, ‘inside’ strategies targeting government actors include private meetings and responding to formal consultations, while ‘outside’ strategies include campaigns, petitions and protests [14]. We apply this typology to generate a framework of strategies, as identified in the international literature, used by NGOs in the commercial determinants of health.

## Methods

A narrative review of peer-reviewed literature was selected due to the primarily interdisciplinary focus, covering political science, public health, policy studies, international relations, business studies and economics [17]. Following international practice, the review involved a) a systematic search of relevant scholarly literature using structured search terms, b) screening of articles by inclusion and exclusion criteria, and c) analysis and thematic synthesis [18, 19].

### Search strategy

A systematic search of three comprehensive databases: Web of Science, ProQuest and Scopus, was conducted on March 9 2022. The search strategy was developed in consultation with two librarians specialising in public health and humanities respectively, both based at the first author’s institution. To identify literature examining NGO interactions with industry and/or government, we selected search terms for three concept categories: commercial determinants of health, governance, and nongovernment organisations. Key terms were selected and refined based on initial scoping of common terms in articles that we would expect to see included in the study. Further terms were added in consultation with librarians (See Table 1). Terms for NGOs were tested through a wide range of search terms, and were narrowed for scope and feasibility to focus on studies that reported on non-government organisations. We limited the review to 1 January 1980 onwards to include the development of global civil society and health policy following the highly relevant landmark Infant Code of Marketing of Breast-milk Substitutes [20].

**Table 1:** Search terms

Category	Search terms
Commercial determinants of health	(Alcohol* OR tobacco OR “tobacco control” OR “non-communicable disease*” OR nutrition OR obesity OR “processed food” OR “infant formula” OR soda OR “sugar sweetened beverage” OR “SSB” OR “unhealthy food OR drink” OR “salt OR sodium reduction” OR “fossil fuel” OR mining OR pharmaceutical OR firearm OR gun OR NRA OR “national rifle association” OR gambling OR pesticide OR agrochemical OR petroleum OR coal OR oil OR gas OR “commercial determinants of health” OR corporat* or industry or TNC)
Governance	(Advocacy OR “Agenda setting” OR attention OR engagement OR frames OR framing OR Priorit* OR commitment OR enable OR constrain OR capacity OR influenc* OR negotiat* OR policy mak* OR process* OR govern* OR polic* OR politic* OR problematisation OR consult OR regulate OR monitor OR accountability OR campaign or partnership)
NGO actors	(“non government* organisation” OR NGO OR “civil society”)

### Selection criteria

**Fig. 1:**
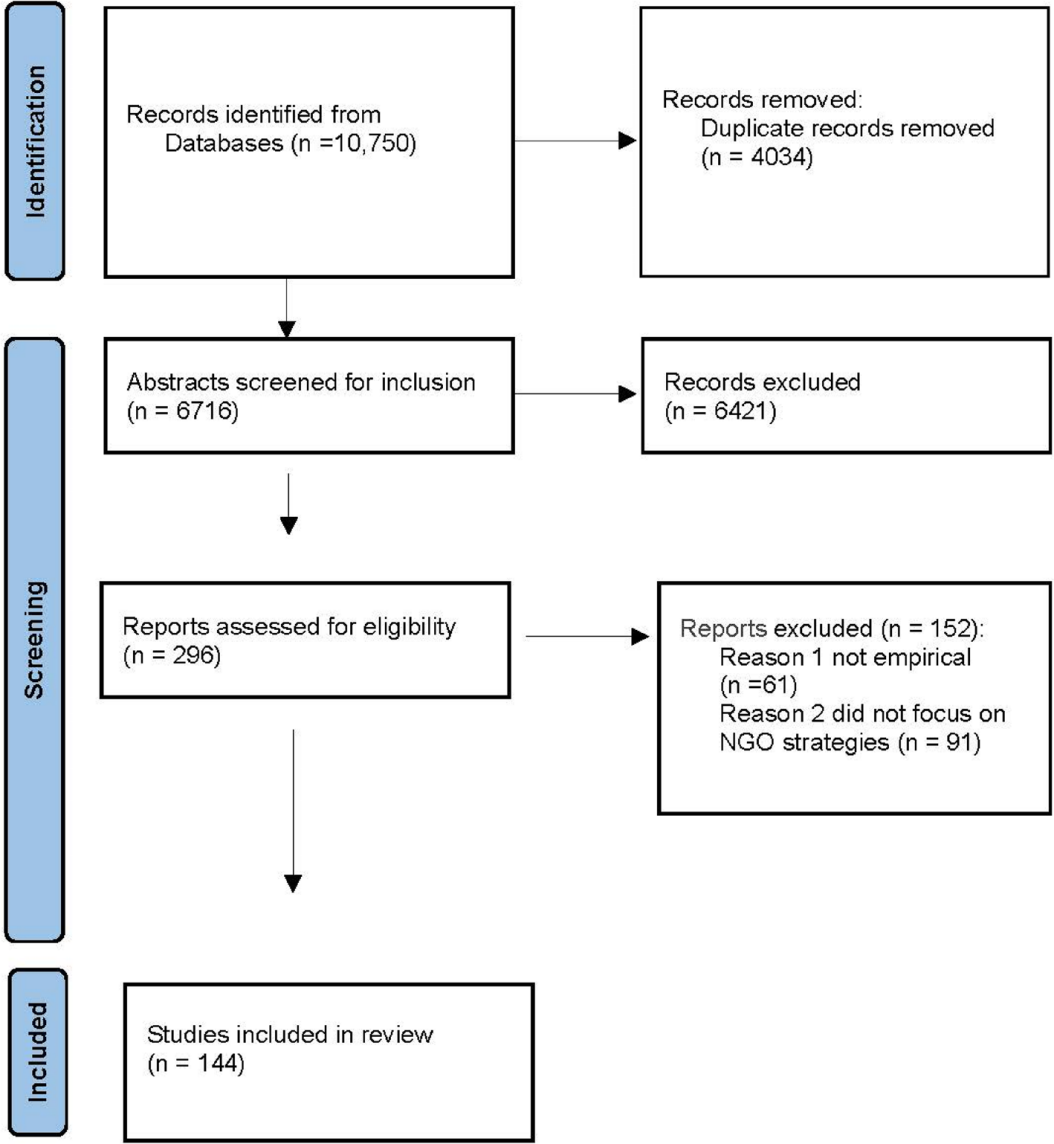
Flow diagram of search

The results of the search string were uploaded into Covidence review software and duplicates removed. Abstracts were screened according to the inclusion and exclusion criteria. Studies were included if they were in English, were peer reviewed, comprised an empirical study (i.e. not an editorial or commentary), explicitly sought to examine ‘NGOs’ (as reported by the authors), and identified at least one strategy aimed at industry and/or government policy and practice. Full text studies were excluded if they did not meet the criteria. Studies were assessed for quality based on the appropriateness of study design, identification of data sources, clarity of findings and justifiable outcomes. Following title and abstract and full-text screening, a total of 144 articles were included in the analysis. A flow diagram of the screening results is outlined in Fig. 1.

### Analysis

Three authors initially screened a set of 40 abstracts to refine the inclusion and exclusion criteria. To assist with thematic synthesis of the strategies used by NGOs in the commercial determinants of health, all included articles were coded in Nvivo qualitative software. Coding was inductive, with like codes grouped together as they emerged according to the framework of inside/outside and commercial / government [14], as well as coding for when studies reported on NGO impact. During the coding of the studies, the author team held a coding workshop to refine the codes and emerging themes. Data about the article characteristics were also extracted from the included studies and collated in Microsoft Excel: including the author(s), title, year of publication, methodology, industry sector, time period of study, NGOs studied, NGO aims, and level of analysis/case country(s) (see Supplementary Table).

## Results

### Industry sectors and study contexts

**Fig. 2:**
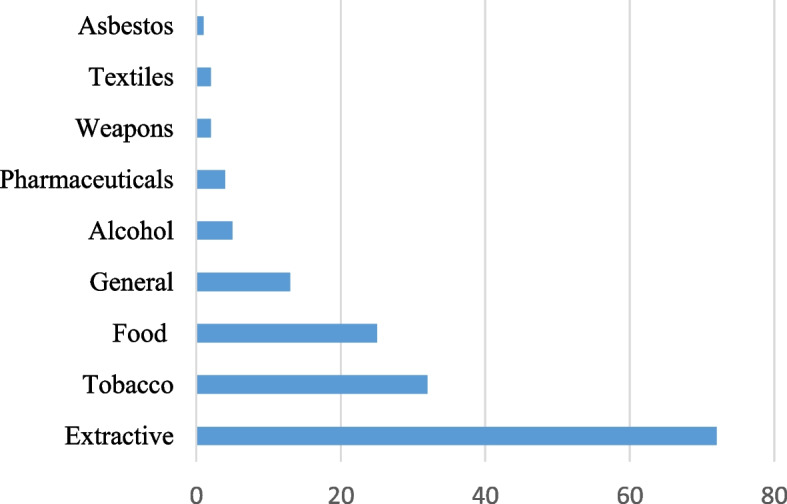
Number of studies, by industry sector

Industry sectors in focus in the studies included eight specific sectors; extractive, tobacco, food, alcohol, pharmaceuticals, weapons, textiles and asbestos (see Fig. 2). We labelled 13 studies ‘general’ as they each encompassed a range of industry sectors. One hundred and thirty-five studies were qualitative, two studies were quantitative, and seven studies used mixed methods.

**Fig. 3:**
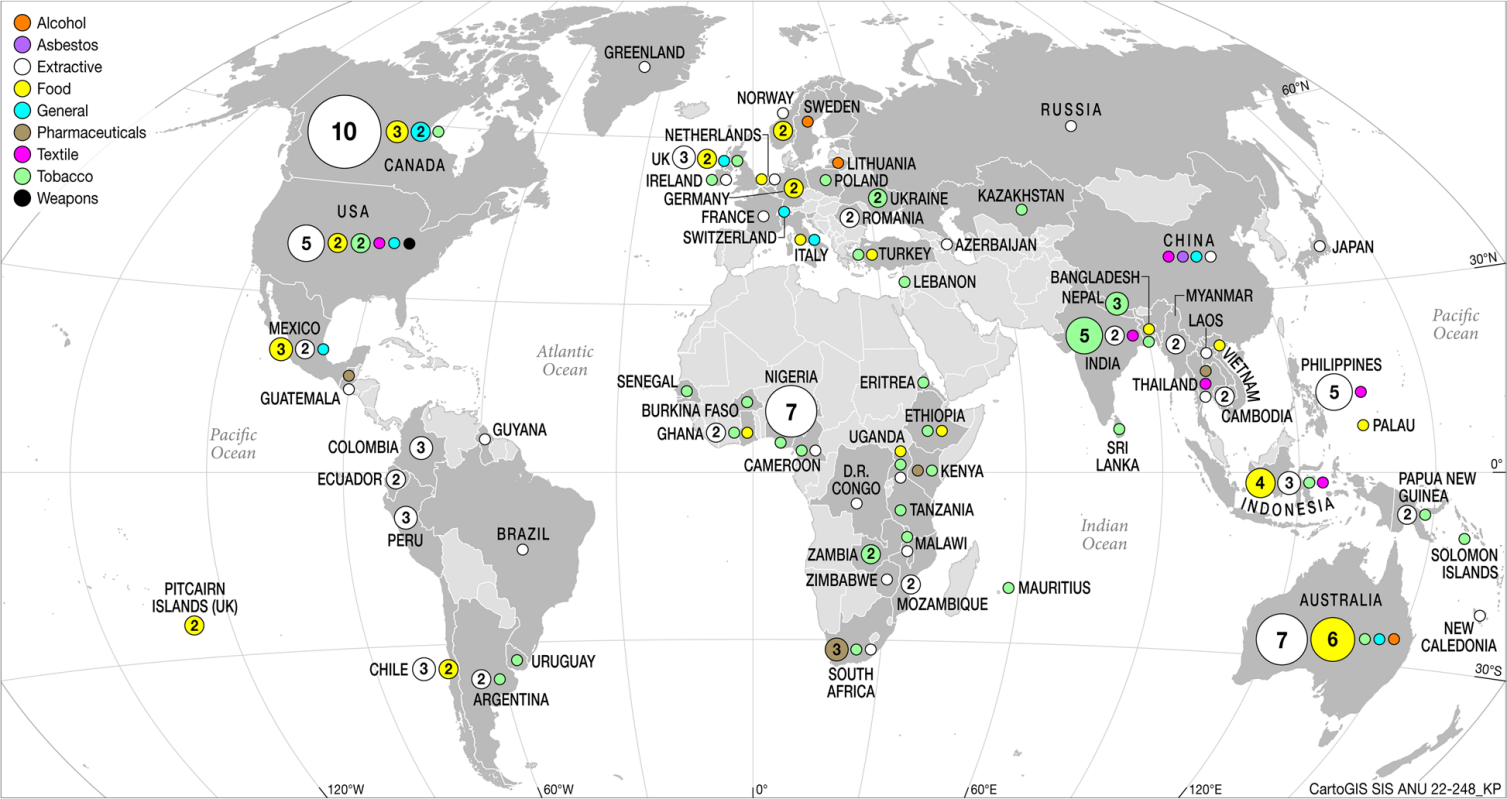
Geographical spread of country studies’ focus

The majority of studies focused on NGO interactions at the country level (79%), followed by the global (19%) and regional levels (2%) (See Supplementary Table). There was a geographic spread for country-level studies in the extractive, tobacco and food sectors across Africa, Asia, Latin America and Europe (see Fig. 3).

Of the 72 country studies in the extractive sector, 10 were in the US, 7 in Canada and 7 in Nigeria. India (5) and Nepal (3) were the most prevalent country studies for tobacco sector (*n* = 32), as were Australia (6), Indonesia (4), USA (3), Mexico (3), Canada (3) in the food sector (*n* = 25). Pharmaceutical studies at the country level included South Africa (*n* = 3), Guatemala (*n* = 1), Kenya (*n* = 1) and Thailand (*n* = 1), and textile country-level studies included one study on Ghana and India and one study on China, Indonesia, the Philippines, Thailand and USA. Alcohol country level studies focused on Australia (*n* = 1), Lithuania (*n* = 1) and Sweden (*n* = 1); asbestos in China (*n* = 1); and weapons in the US (*n* = 1) (see map Fig. 3).

#### Framework of NGO strategies in the CDOH

We identified 18 different ‘inside’ and ‘outside’ strategies used by NGOs across sectors to target commercial or government actors (see Table 2). We explain each of these strategies below.

**Table 2:** Framework of NGO strategies in the CDOH

	**Target: Commercial actor**	**Target: Government / Intergovernmental**
Inside strategies	• Forming partnerships and multistakeholder initiatives • Holding private meetings and roundtables • Engaging with company AGMs and shareholders • Collaborating other than a partnership; including via the participation in the development of industry standards, reports, CSR initiatives • Pursuing litigation directly against industry	• Lobbying; including via formal consultation processes (e.g. submissions), serving on committees and in delegations, and informal interactions with policymakers • Drafting legislation, policies and standards; including co-producing reports • Providing technical support and training; including funding government activities and taking on implementation roles • Pursuing litigation against government
Outside strategies	• Monitoring and reporting on commercial practises; including developing ‘counter accounts’ to debunk industry claims, calling out industry funded groups, ‘naming and shaming’ in mainstream media and social media campaigns • Protests at industry sites • Boycotts • Directly engaging with the public in campaigns, including mass letterboxing and coordinating mass petitions • Creative use of alternative spaces; including via public documentaries of corporate activities	• Protests and public campaigning calling for regulation; including via mainstream media and social media • Monitoring and reporting, including monitoring government compliance with international treaties and generating evidence for monitoring • Forum shifting debate from one policy forum to another – vertically within government and horizontally to the global level • Proposing and initiating alternative sources of economic development

Matrix adapted from Colli and Adriaensen [14]

#### ‘Inside’ strategies directly targeting commercial actors

We identified from the studies five types of strategies used by NGOs to directly target commercial actors: 1) partnerships and multistakeholder initiatives; 2) private meetings and roundtables; 3) engaging with company AGMS and shareholders; 4) collaborations other than partnerships, and 5) litigation. 58 studies (40%) reported at least one NGO inside strategy directly targeting commercial actors.

NGOs sought to influence commercial actors directly through the formation of partnerships and multistakeholder platforms – a strategy identified in 18 studies [21–38]. Partnerships included between the World Wildlife Fund and Unilever [28, 33], Coca Cola, Nokia and Hewlett-Packard [34], and environmental NGOs partnerships with British American Tobacco [30]. Partnerships also comprised NGO-commercial-government (and thus encompassed ‘inside’ strategies with government). Five studies examined the monitoring role of NGOs in the Extractive Industries Transparency Initiative (EITI), a partnership between NGOs, commercial actors and governments at the country-level designed to promote transparency in extractive industry governance [27, 29, 35–37]. In several studies, engaging in partnerships were reported to be an incremental strategy by NGOs as part of a longer-term campaign for greater regulation of industry practices [22, 29, 35, 36], although some studies found that these partnerships enabled corporate actors to have greater influence on policymaking [30].

A second NGO ‘inside’ strategy was holding private meetings and roundtables with commercial actors, also identified in 18 studies [22, 23, 32, 33, 38–51]. Private meetings have been used by NGOs to attempt to find commercial allies – for example through the formation of private NGO-industry roundtables on reducing fossil fuel emissions [32]. NGOs have also used meetings to target ‘surrogate’ commercial actors in supply chains. Four studies, for example, explored NGOs using private meetings with banks and investors to attempt to influence loans made to extractive companies [33, 48–50]. While the majority of studies did not analyse the content of NGO-commercial actor meetings in depth, one study of NGO engagement with extractive industries documented the development of NGO-industry terms of engagement [45].

The third NGO ‘inside’ strategy was engaging with company shareholders, identified in 14 studies [23, 33, 42–44, 48, 50, 52–58]. In the majority of these studies, NGOs spoke at company annual general meetings and company board meetings, and targeted shareholders and investors through statements and shareholder resolutions in an attempt to influence corporate behaviour directly [23, 42–44, 48, 52–56, 58]. Two studies outline further NGO engagement, including briefing, consulting and advising shareholders, becoming shareholders themselves, and creating mutual funds to influence a range of investors [55, 56]. Four studies explored a transnational dimension to shareholder engagement through which NGOs from low income countries used their networks to speak at shareholder and company meetings in the US [57], Canada [23], Australia [50], and France [33].

A fourth NGO ‘inside’ strategy was collaborations other than formalised partnerships, identified in 13 studies [21, 22, 45, 53, 59–67]. Commercial actor-NGO collaborations included collaborations on sustainability standards [62, 64], reducing food waste (for example, between NGOs and McDonald’s) [59], participation in CSR initiatives [66] and collaborating on reports – for example reports produced by Oxfam and Unilever [22]. Informal collaborations between NGOs and media organisations were documented in 4 studies, where NGOs provided training to journalists of media organisations on monitoring tobacco industry practices [60, 61, 67] and childhood nutrition and infant feeding [68].

Finally, NGO litigation directly against a commercial actor was a common strategy in 20 studies [22, 23, 41, 48, 50, 52, 69–82]. Unlike litigation against government (see below), this strategy focused on litigating directly against an commercial actor. In South Africa, NGO litigation against pharmaceutical firms over lack of access to HIV/AIDS treatment has resulted in the companies being found guilty of abusing antitrust law [77]. In India, NGOs filed public interest cases against mining giant Rio Tinto on charges of illegal mining, leading to temporary moratoriums on mining practices [78]. Litigation has also been used by NGOs against media companies and advertising firms over the broadcasting of cigarette advertising [82]. Three studies reported on a transnational dimension to NGO litigation where networks filed suits where TNCs were headquartered – for example litigation against Shell in the UK [75, 76] and US [76], and in Canada against transnational mining firms on behalf of NGOs in Latin America and Africa [79]. We classified litigation as ‘inside’ because it represents an attempt to directly influence a commercial actor, though this strategy can include outside elements (in generating public attention), and relies on the state apparatus (i.e. the court system).

#### ‘Outside’ strategies targeting commercial actors

Five types of NGO ‘outside’ strategies targeting commercial actors were identified across the studies; 1) monitoring and reporting on commercial practises; 2) protests at commercial actor sites; 3) boycotts; 4) directly engaging with the public; and 5) creative use of alternative spaces. 83 (58%) of studies explored at least one NGO ‘outside’ strategy directly targeting commercial actors.

The most prevalent NGO ‘outside’ strategy targeting commercial actors was monitoring and reporting on practises harmful to health – identified in 56 studies [21, 22, 26–28, 31, 33, 38, 40, 42–45, 47–49, 52–54, 58, 67, 72, 73, 75, 78, 81, 83–106]. NGO monitoring included the development of ‘counter accounts’ to challenge industry claims and provide alternative evidence on impacts to health [31, 33, 43, 47, 54, 72, 78, 83, 92–94]. NGOs in Nigeria, for example, developed the Oil Spill Monitor (OSP) to document oil pollution, impacts, and extractive industry remediation [43, 95]. NGOs used ‘naming and shaming’ campaigns in mainstream media in 10 studies [22, 38, 48, 52, 53, 67, 85, 99–101], and social media campaigns to target commercial actors in 12 studies [42, 47, 54, 58, 75, 88, 101–106]. For example global NGO Greenpeace has used social media campaigns to target Nestle [106], Apple [88] and ExxonMobil [47].

Five studies documented NGO monitoring of industry interference in policymaking [40, 94–97]. One study, for example, reported on NGO monitoring of tobacco industry activities during the FCTC negotiations [97]. Two studies documented NGO monitoring and reporting on industry-funded front groups [84, 98]. This strategy was reported to be successful in Australia, with NGOs targeting pro vaping groups for their tobacco industry links, limiting their influence on policymaking [98]. Subsequent to monitoring, eleven studies reported on NGOs then developing their own standards of conduct and guidelines to publicly shame commercial actors to improve their practices [22, 23, 25, 45, 52, 53, 62, 64, 65, 86, 107].

Holding protests at industry sites was identified in 24 studies [22, 26, 29, 41–44, 47, 49, 53, 57–59, 64, 71, 72, 77, 78, 86, 89, 106, 108–110]. Protests draw public and media attention, and have been used by Greenpeace against Nestle for using uncertified palm oil [106], and by the NGO network Treatment Action Campaign against pharmaceutical giant Pfizer [77]. NGO protests have included occupying industry sites [44, 58, 64, 108], blocking entry to an industry site [26, 86], breaking into sites e.g. breaking into McDonald’s [59], and publicly disrupting company AGMs and shareholder meetings [47, 106].

Third, the use of boycotts, including consumer boycotts and targeting other commercial actors in the supply chain, was a strategy identified in 13 studies [22, 47, 52, 53, 59, 63–65, 86, 88, 111–113]. Most of the studies examined a consumer boycott, for example campaigns asking consumers not to use a particular extractive product [63]. A smaller number of studies identified a boycott strategy focused on commercial actors in the supply chain – for example pressuring grocers and schools not to sell particular unhealthy products [22, 59] and pressuring Facebook to shift its operations to renewable energy [88].

Fourth, NGOs directly engaged with the public to influence commercial actors. 5 studies documented the use of public letterboxing as a tactic to shame commercial actors though campaign material [52, 53, 58, 102, 114], and one reported on the creative use of a ‘send back’ campaign coordinating mass mailing of McDonald’s packaging by the public to national headquarters [59].

Finally, NGO’s creative use of alternative spaces was an ‘outside’ strategy identified in 26 studies [32, 37, 43, 48, 52, 53, 57–59, 70–72, 79, 81, 90, 92, 101–103, 109, 114–119]. Eight studies reported NGOs producing public documentaries that exposed corporate activities [43, 48, 57, 103] and public goods [32, 90, 109, 115]. Two studies documented the formation of an alternative International People’s Health Tribunal by a network of NGOs against an extractive industry [70, 79], the outcomes of which were then presented through subsequent inside strategies in meetings with politicians and company shareholders (see more on dual strategies below) [70]. In Argentina, NGOs installed a tent as an alternative meeting space in protest against extractive projects [81]. NGOs held alternative climate justice conferences nearby to United Nations climate change negotiations [118]. One study documented the development of community art projects to create space to target the tobacco industry in Indonesia [92].

#### ‘Inside’ strategies targeting government and/or intergovernmental actors

We identified four types of NGO ‘inside’ strategies targeting government and/or intergovernmental actors across the studies; 1) lobbying; 2) drafting legislation, policies and standards; 3) providing technical support and training; and 4) litigation. 84 studies (58%) explored at least one NGO ‘inside’ strategy directly targeting government and/or intergovernmental actors.

The most prevalent NGO ‘inside’ strategy targeting government and/or intergovernmental actors across the studies was lobbying policymakers, identified in 54 studies [13, 23, 26, 29, 32, 36, 40–42, 44, 48, 52, 61, 71, 72, 78, 82–85, 89, 93, 97–100, 103, 108, 109, 112, 114–116, 118, 120–135]. NGO lobbying in the literature included through formal policy processes such as submissions [13, 32, 89, 93, 98, 99, 130, 132, 134, 135], and informal processes such as through hallway corridor conversations with policymakers [97, 128, 132, 133]. One study reported, for example, on how NGOs in Lebanon formed an informal parliamentary friends committee for parliamentary allies of tobacco control [108]. Seven studies explored NGOs serving on government committees as a strategy to influence regulatory outcomes [21, 23, 57, 112, 119, 134, 136]. Three studies documented NGO providing private screenings of their documentaries for policymakers and politicians in an attempt to influence regulation [85, 115, 116]. While a majority of the studies reported on NGO lobbying at the national level, four studies explored NGO lobbying at the intergovernmental level, including lobbying delegates at global climate change negotiations [118], the FCTC [97, 133] and WHO [128].

NGOs were also involved in drafting legislation, policies and standards for government actors in 20 studies [13, 37, 40, 61, 68, 83, 90, 91, 111, 119, 120, 134, 135, 137–139]. NGOs in Myanmar, for example, helped draft the Environment Law and Environment Impact Assessment procedures by government, and contributed to Myanmar’s submissions on international climate change negotiations [119]. In India, NGOs drafted a Code of Conduct at the subnational level on engagement between tobacco industry and government officials [137]. In the Philippines, NGOs have drafted mining moratoriums with local government actors [139], and in Bangladesh NGOs have drafted nutrition policy on infant breastfeeding [68]. In India, NGOs were tasked with drafting a roadmap for local implementation of tobacco control [138]. In Russia, Greenpeace and local NGOs collaborated with government officials on developing a monitoring databases for oil leaks [49]. Four studies explored NGO roles in developing standards and reports [21, 85, 115, 138].

The third NGO ‘inside’ strategy targeting government and/or intergovernmental actors was the provision of technical support and training, found in 20 studies [13, 40, 60, 61, 68, 91, 96, 97, 100, 114–116, 118, 133, 138, 140–143]. Twelve of the total 20 studies exploring this NGO strategy were focused on tobacco control in low-income countries [40, 60, 61, 91, 96, 100, 114, 138, 140–143]. In India, NGOs have run state-based workshops to sensitise government officials on the FCTC and provide technical support [96, 138, 140]. Similarly, a transnational network of NGOs assisted Columbian officials with training, education, and legal defence for implementation of tobacco control regulation in the face of industry opposition [141]. In Uruguay, government health officials requested local NGO support on FCTC legal implications, who in turn brought in US based NGOs to provide advice and financial support [100]. In Turkey, NGOs have run workshops for government officials to counter tobacco industry interference in the development of tobacco control legislation [142]. Likewise, NGOs in Bangladesh have provided technical support and training on child nutrition and breastfeeding to government officials [68]. Three studies explored the role of Canadian and Australian NGOs in providing technical support to low income country delegations in the FCTC [13, 97, 133], and one in global climate change negotiations [118] and one in negotiations to ban land mines [129]. Beyond technical support and training, three studies reported on well-resourced NGOs providing funding to low income countries; for monitoring in the Pitcairn Islands [115], establishing a trust fund for alternative revenue in Kiribati [116], and sponsoring delegations to participate in the FCTC negotiations [13].

Finally, NGOs targeted government and/or intergovernmental actors directly through litigation in 22 studies [23, 27, 33, 40, 72, 74, 77, 79, 81, 82, 85, 87, 90, 114, 124, 137, 139, 144–148]. In these studies, NGOs targeted government rather than industry as above. In Indonesia, for example, NGOs successfully litigated in the Supreme Court for public disclosure of mining licenses [27]. In Australia, one NGO successfully used litigation which forced the federal government to set aside environmental approval for a mine [147]. In India, public interest litigation has been used by NGOs at the subnational level to require implementation of tobacco control legislation [137].

#### ‘Outside’ strategies targeting government and/or intergovernmental actors

Sixty-eight studies (47%) explored at least one NGO ‘outside’ strategy targeting government actors. We identified four NGO ‘outside’ strategies classified as; 1) protests and public campaigns; 2) monitoring and reporting; 3) forum shifting; and 4) proposing and initiating alternative solutions**.**

The most prevalent NGO ‘outside’ strategy targeting government and/or intergovernmental actors was the use of protests and public campaigns calling for greater government intervention and regulation, identified in 43 studies [13, 29, 32, 40, 41, 51–53, 58, 60, 64, 77, 78, 83, 88–91, 99, 101, 103, 104, 108, 110, 112, 114, 118, 119, 121, 122, 124, 126, 129, 130, 138, 139, 141, 145, 148–152]. Two studies reported on transnational protests through the International Day of Action on Climate Change [32, 118]. Three studies explored NGOs use of open letters to government as part of wider campaigns in extractive [119], alcohol [51] and corporate tax avoidance [89]. NGO protests included occupying government sites [88, 145], parliament [108], and disrupting global negotiations [118]. The symbolic use of a ‘death clock’, a digital clock displaying worldwide deaths from tobacco and hung at the entrance of the FCTC negotiations by NGOs, was identified as an influential symbolic tactic that framed the need for tobacco control as a global public health issue [13]. Public campaigns included through mainstream media [29, 32, 40, 53, 60, 83, 91, 99, 103, 104, 108, 112, 114, 121, 122, 124, 129, 138, 141, 149, 150] and through social media [32, 88, 89, 101, 118, 130], with government as the specific target. One study, for example, documented the use of shaming awards in media publications by the global Framework Convention Alliance as a key tactic to influence government positions during the WHO FCTC negotiations [13].

Monitoring and reporting was a key strategy identified in 23 studies [29, 32, 36, 37, 40, 43, 51, 61, 75, 81–84, 86, 93, 97, 121, 134, 136, 147, 148, 150, 153]. Unlike monitoring industry actors, as outlined above, this tactic focused exclusively on government. Fourteen studies explored NGOs developing reports of government regulatory initiatives that were designed to focus public attention [29, 32, 36, 37, 43, 75, 81, 93, 121, 134, 136, 147, 150, 153]. For example, NGOs monitoring government compliance with the FCTC [61, 83] and International Code on Breastmilk Substitutes [86]. Three studies reported on NGOs using scorecards [43, 61, 134], and two studies reported on NGO monitoring of government engagement in multilateral negotiations, including the FCTC [97] and the WTO Doha Declaration [148]. As a subset of monitoring, four studies explored NGO monitoring of linkages between government and industry actors [40, 51, 82, 84]. For example, one study explored NGOs publicising tobacco industry donations to the then Nepalese Health Minister [40]. In another study, NGOs in the EU exposed tobacco industry and government official ties [84]. In Lithuania, NGOs published a website targeting MPs who voted for cancelling a proposed alcohol advertising ban [51]. Furthermore, different types of evidence were generated by NGOs as part of monitoring and campaigning (see Table 3).

**Table 3:** Evidence generated by NGOs in monitoring and campaigning

• Scientific studies [21, 22, 59, 83, 115, 116, 120, 136]. For example, NGOs in India conducted a study of Coca Cola products, identifying pesticide residue, informing a campaign against the company [22]
• Human Rights Impact Assessments [23]
• Specific health evidence [60, 83–85, 108, 121, 122, 149]. For example, an NGO produced a study of cancer risks from a company pesticide, informing a boycott and media campaign [59]
• Purchasing power studies [52]
• Public opinion polling [39, 61, 83, 121, 149]
• Economic evidence [83, 115, 121, 122]
• Personal stories [69]
• Local evidence—highlighted as important to support transnational NGO campaigns on the ground [108]
• One study comparing the success and failure of different NGO campaigns asserted that success was due to presenting evidence as legitimate knowledge, and in particular epidemiological data as a source of legitimate knowledge [9, 111, 154–157]

A third NGO ‘outside strategy’ targeting government actors was forum shifting – where NGOs strategically shifted debate from one policy forum to another in an attempt to obtain favourable reception and influence. This strategy was identified in 17 studies [23, 36, 48, 49, 52, 61, 71–73, 83, 84, 107, 109, 128, 145, 158, 159]. Most studies explored NGOs forum shifting vertically, from the national to regional and global level [23, 36, 48, 49, 52, 61, 71, 72, 83, 84, 107, 109, 128, 158]. Three studies examined NGOs forum shifting to UN committees to shame Nigeria [109], Russia [49], and Ghana [48] on extractive governance (found to be instrumental in Ghana withdrawing military protection for mining companies) [48]. Three studies explored NGOs forum shifting to the FCTC Conference of Parties to shame high and low income country governments for lack of FCTC compliance [61, 83, 84]. One study explored NGOs forum shifting to the United Nations Convention on the Elimination of All Forms of Discrimination Against Women (CEDAW) Committee to frame tobacco control as an issue for women’s rights, successfully influencing the adoption of tobacco control legislation in Argentina [158]. Regionally, NGOs filed labour violation complaints through the North American Free Trade Agreement [52]. Four studies explored NGOs forum shifting horizontally, to other government departments [23, 48, 73, 145]. Two studies explored NGOs in Mexico [73] and India [145] shifting the issue of extractive governance to their respective national Human Rights Commissions. NGOs have also shifted transnationally to other parliaments. One study, for example, explored NGO networks enabling Philippine NGOs to present to the Canadian Parliamentary Subcommittee on Human Rights and Democratic Development regarding Canadian mining company practices in the Philippines [23].

Finally, seven studies explored NGOs proposing and initiating alternative solutions for economic development as an ‘outside’ strategy targeting government [37, 71, 72, 101, 103, 116, 119]. Through this strategy, NGOs publicly proposed alternative solutions to harmful commercial practices to government as a strategy to convince the public of alternative sources of revenue to commercial practices. NGOs working to reduce reliance on extractive industries in Myanmar, for example, developed small-scale renewable energy projects to demonstrate to the public and government that there were alternative sources of employment and income other than extractive projects [119].

#### Dual strategies

A majority of the studies (78%) identified more than one NGO strategy, yet few studies explicitly noted combinations of ‘inside’ and ‘outside’ approaches nor identified different approaches for different targets (e.g. government or commercial actors). Two studies documented NGOs working inside the FCTC negotiations as part of Canada’s official delegation, while simultaneously working outside the negotiations calling for strong tobacco control provisions [97, 133]. Two studies reported on NGOs participating in ‘inside’ multistakeholder initiatives while simultaneously engaging in outside strategies to call on commercial actors to improve their practices [27, 28]. One study explicitly reported on NGOs strategically targeting both commercial and government actors through monitoring and lobbying [81]. One study of tobacco control in Indonesia reported on NGOs working through inside lobbying and outside campaigning to publicly exposing links between government actors and the tobacco industry [82]. Interestingly, one study was designed to assess and compare the success of NGO inside and outside strategies targeting an commercial actor (in this case grocers in Sweden for reducing alcohol sales to minors) [38]. The study found that the outside tactic – public shaming of the grocer, was four times more effective than the inside tactic of dialogue in reducing alcohol sales to minors [38].

#### Conceptualising NGO impact in the CDOH

In addition to mapping NGO strategies, as outlined above, we sought to identify how NGO impact was conceptualised in the literature. We were unable to assess the impact of particular NGO strategies, as most of the literature did not report this. Rather, we were able to identify that NGO impact was spoken about in the literature (in 43 of the studies or 30% of studies) in three ways: substantive, procedural, and normative (see Table 4).

**Table 4:** Examples of NGO impact

Substantive	Procedural	Normative
NGO collaboration with state officials at the subnational level in India was key to several Indian states notifying implementation of FCTC Article 5.3, particularly on limiting government-tobacco industry interactions [96] NGOs successfully pressured a newspaper company in the US to ban advertisements of particular weapons [85] and pressured banks not to finance new extractive industry projects in Australia [129, 160] NGO secured amendments to parliamentary bills to include compensation for communities affected by mining [48] NGOs successfully pressured mining companies to withdraw license applications [22, 57] NGO litigation has secured toxic clean-up, health and safety improvements, wage raises, and financial payment of damages [52, 76] International NGO shaming of government had a negative effect on foreign investment flows into the shamed state, indicating commercial actors are sensitive to reputational damage [113] NGO’s successfully lobbied Hong Kong to amend policy to incorporate mesothelioma as a disease for compensation [127] NGO boycott campaigns successfully led to Uniroyal ceasing production of a pesticide [59]	NGOs secured participation in processes and greater transparency in processes e.g. transparency in reporting of extractive resource income [27, 36, 93, 117] NGOs secured improvements to future government EIA processes [81] In 2018, legal action by a local NGO in Mexico against a mining company led to a Supreme Court ruling that local communities had the right to participation and consultation in projects that affected their right to a healthy environment—establishing a right to consultation for future enforcement [73] In Ghana, NGOs successfully blocked industry representation on the governing body of oil and gas production, instead securing civil society representation [134]. In Lebanon, NGOs used the FCTC to successfully campaign for industry actors to no longer be included in formal deliberations by parliamentary committees [108] Public What You Pay Indonesia used its participation in a multistakeholder platform to shape processes to improve extractive industry governance, while litigation outside the platform by other NGOs secured processes for the public disclosure of mining licenses [27] In Kerala, India, local officials shut down a Coca Cola bottling plant after an NGO campaigned that it drained and polluted local water [22]	Two studies attributed the global diffusion of a marine protection norm, which has informed the development of multiple marine protection areas free from industry interference, to the norming diffusion strategy of global NGOs Pew Trust and National Geographic Society [115, 116] One study found the impact of NGO movements on shifting discourse towards food sovereignty and sustainability in Canada. [117] In another study, while unsuccessful in stopping extractive projects, transnational NGO networks were found to have elevated framing of human rights and concerns about Indigenous people’s rights in the context of extractive industry practices at the global level [49] NGO worker exchanges between NGOs in Canada, Mexico and USA (on behalf of workers employed by the same company) have shifted local frames from nationalist to international labor rights and global solidarity framing [52] A study of NGO campaigning on tax justice in Australia found that the NGO campaign successfully influenced the public narrative, framing corporate tax avoidance as a “revenue” problem”, which was taken up by the Government and media [89]

Twenty-five studies (17%) attributed NGOs as making a *substantive* impact – that is, they significantly influenced commercial practices or government/intergovernmental policy and regulation [22, 26, 40, 44, 48, 52, 57, 59, 76, 77, 85, 96, 106, 108, 111, 113, 115, 124, 127, 129, 134, 141, 146, 158, 160] (see Table 4). A further 13 studies identified a partial impact on policy and regulation, in which NGOs temporarily influenced policy, such as halting mining licenses, or industry responded through voluntary actions [23, 27, 49, 61, 70, 72, 77, 79, 83, 89, 90, 135, 139, 143].

The second type of NGO impact we identified was *procedural* impact, that is, influencing processes and procedures such as securing representation on committees, greater transparency, or preventing industry representation, identified in 12 studies (8%) [22, 27, 36, 73, 81, 87, 90, 93, 108, 117, 133, 134]. The third type of NGO impact was *normative* impact, where NGOs were identified as having successfully diffused their particular framing in policy, media or corporate discourse, identified in nine studies (6%) [36, 49, 52, 89, 104, 115–117, 161] (see Table 4).

## Discussion

Calls for a ‘public health playbook’ point to the need for public health actors to develop a suite of strategies to counter corporate power and influence in the commercial determinants of health [7, 162, 163]. NGOs are one important group of actors who actively seek to counter commercial practices. Our review, based on a systematic search of peer-reviewed literature across a range of disciplines, identified 144 studies of NGO activities across a range of industry sectors and at different levels of governance. We identified 18 inside or outside strategies that have been used by NGOs to target government or commercial actors.

Half of the studies focused on NGO activities in the extractive industry sector, followed by tobacco (22%), food (17%), and general (i.e. the target was a range of sectors, 9%), with the remaining 2% capturing NGO activities specifically related to alcohol, pharmaceuticals, weapons, textiles and asbestos. The dominance of the extractive sector in the included studies may be explained by a clear focus of policy studies literature on environmental NGOs in comparison to other health issues. Many established environmental NGOs have a long history of political campaigning, particularly since the 1992 Rio Declaration on Environment and Development, which may also explain why this sector was dominant. We were surprised to see fewer studies on NGO tactics in other sectors, which were included in our search terms (i.e. none on NGOs in gambling), which remains a limitation of the review (see more on limitations below). It is also worth noting that many of the NGOs in the extractive sector captured in the included studies are not necessarily “health” NGOs (see Supplementary Table 1 for the names of NGOs listed), nevertheless these studies can provide important lessons for public health in terms of the suite of strategies identified.

A majority of the included studies (79%) examined NGO activities at the country level, followed by the global (19%) and regional levels (2%) (See Supplementary Table 1). There was a geographic spread of studies for the extractive, tobacco and food sectors across Africa, Asia, Latin America and Europe (see Fig. 3). One explanation for the dominance of country studies is that NGOs may have greater access to policy processes at the national level than at the global or regional level. Another is that generating public attention may be easier when focusing on domestic issues (or many national NGOs naturally focus on advocacy at the national level). Another reason is that country level studies may be easier to fund and research. An outlier is the tobacco set of studies, several of which reported on NGO interactions at the global level, including infiltrating government delegations and using ‘inside’ and ‘outside’ tactics focused around the WHO FCTC [40, 60, 61, 91, 96, 100, 114, 138, 140–143]. This suggests that a global instrument like the FCTC can serve as a focal point or catalyst for NGO activities at the global or regional level.

Our analysis of the 144 included studies identified five NGO ‘inside’ strategies directly targeting commercial actors: 1) participation in partnerships and multistakeholder initiatives; 2) private meetings and roundtables; 3) engaging with company AGMs and shareholders; 4) collaborations beyond partnerships, and 5) litigation. These ‘inside’ strategies appeared to sit on a spectrum from collaborative to conflictual, with a common theme being direct engagement with the commercial actor in an attempt to influence change in practice. Of the partnerships identified, the majority were between environmental NGOs and industry actors, such as World Wildlife Fund partnering with Unilever [28, 33], Coca Cola, Nokia and Hewlett-Packard [34]. Engaging in partnerships was reported to be an incremental strategy by some NGOs in the studies as part of a longer-term campaign for greater regulation of industry practices [22, 29, 35, 36]. One study of British American Tobacco (BAT) and an environmental NGO, however, found that this partnership enabled BAT to have a greater influence on government policymaking [30]. It is also important to note that some multistakeholder initiatives comprised government and commercial actors (and are therefore captured as strategies in both quadrants of the matrix).

The majority of studies documenting engagement with company AGMs and shareholders were in the extractive sector, and included collaborative and conflictual activities such as speaking at AGMs, lobbying investors, briefing advisers, and becoming shareholders [23, 33, 42–44, 48, 50, 52–58]. Litigation was classified as inside due to the target (i.e. commercial actor), acknowledging there can be an outside element to this strategy and a reliance on court systems. This strategy of litigation highlights the fuzziness of the heuristic – not all activities neatly fit within one quadrant of the matrix. The policy studies matrix has been useful, however, in documenting the range of strategies targeting either government or intergovernmental organisations or commercial actors directly in the CDoH. The findings from applying this matrix framework to CDoH literature indicates that different strategies are used for different actors and different purposes (e.g. inside influence or outside public opinion), which NGOs and other policy actors can use when seeking to influence the CDoH.

Of the NGO ‘inside’ strategies targeting government, lobbying was the most prevalent, followed by; drafting legislation, policies and standards; providing technical support and training; and litigation. Twelve of the 20 studies exploring NGO activities in providing technical support and training to government actors were focused on tobacco control in low-middle income countries [40, 60, 61, 91, 96, 100, 114, 138, 140–143]. This finding indicates that NGOs have acquired technical expertise in tobacco control, including through understanding and translating commitments on the FCTC.

Of particular interest for the NGO ‘outside’ strategies targeting government was the use of forum shifting—where NGOs strategically shifted debate from one policy forum to another in an attempt to obtain favourable influence [23, 36, 48, 49, 52, 61, 71–73, 83, 84, 107, 109, 128, 145, 158, 159]. Most studies explored NGOs forum shifting vertically—from the national to regional and global level [23, 36, 48, 49, 52, 61, 71, 72, 83, 84, 107, 109, 128, 158]. This finding supports Keck and Sikkink’s [164] analysis of the ‘boomerang effect’ in social movement literature, which describes how NGOs experiencing state blockages can manoeuvre to global forums to put pressure on states via alternative routes. Further research on forum shifting can indicate when and how this strategy can be effective for NGOs, particularly when working in the global context.

Finally, it was not unsurprising that most studies did not report on the impact of particular NGO strategies, given the complex nature of the policy process. However, 43 studies did reflect on NGO impact in one of three ways: substantive, procedural or normative (see Table 4). NGOs are reported to not only have a direct influence on policy change, but can influence procedure and process, or the norms and dominant ideas shaping the governance of commercial determinants. Future research is needed to tease out the pathways through which particular strategies, or combinations of strategies, and under what conditions, leads to impact on the CDoH.

There are several layers of potential future research from this analysis, including: why NGOs use particular strategies, which combinations of strategies are effective, and the conditions that can enable NGOs to influence government or commercial actors. Here we can speculate on the conditions that may enable NGOs. *Internal resources* such as adequate funding and capacity are likely an important factor. Funding for NGOs, for example by Bloomberg and Vital Strategies, appeared to be important in some studies to enable NGOs to undertake certain strategies [83, 100, 102]. External factors such as *supportive actors* are also likely important. Indeed supportive leaders and allies were evident in some of the studies [61, 115, 123]. Similarly, *favourable policies and processes* may influence choice of strategy*.* Access to political processes, the presence of international treaties to draw on (such as the FCTC [61]), favourable national legislation, and favourable state and market structures likely influence NGO strategies [14]. It is worth noting that in the case of tobacco, the FCTC precludes tobacco industry-government interactions, which has a flow-on effect for health NGOs not engaging with tobacco firms (although our studies show that some environmental NGOs have partnered with tobacco industry actors). This has implications for wider approaches to partnerships and the management of potential conflicting interests. Better understanding of what works can help inform NGO approaches to managing such tensions. Finally, *contextual factors* like the health context, economic context, public opinion, and financial incentives for government (e.g. taxes create revenue) likely enable or constrain NGO impact, and are worth investigating.

### Limitations

Articles were identified from the structured search, and as a result we may not have captured articles that did not use the key terms selected. While we used keywords for gambling, no studies of NGOs activities in gambling appeared in the search. We focused on NGOs, which relied on the authors of the papers identifying NGOs as their focus of study (i.e. not social movements), and as a result we may have missed papers which used other terms such as ‘advocates’. The majority of studies did not identify the type of NGO. As a result, the strategies are not broken down into sub types of NGOs. As the studies covered a span of decades, the analysis did not lend itself to characterising NGO activities by country income group (as countries have changed in their income status over time). This type of study did not enable a causal identification of which strategies or combinations of strategies lead to impact (or are appropriate) – rather, we are providing a suite of known strategies through which the impact, appropriateness and conditions for influence can be further explored.

## Conclusion

Public health scholarship has uncovered a wide range of strategies used by industry actors to promote sales of their products and to influence government policy and regulation. Less is known about the strategies used by NGOs to influence commercial practices, and how NGO impact is conceptualised. This examination of peer-reviewed literature identified 18 NGO strategies across ‘inside’ and ‘outside’, targeting government’s or commercial actors. In doing so, it offers a suite of strategies, and contributes to the wider policy studies literature on specific tactics from the commercial determinants of health. These can be applied to investigate why and how particular strategies are effective, or appropriate, and under what circumstances and conditions.

### Supplementary Information

**Additional file 1: Supplementary Table.** Included studies.

## Data Availability

The data set analysed during the current study are available in the Supplementary Table.

## Abbreviations

BAT: British American Tobacco
CDoH: Commercial determinants of health
EITI: Extractive Industries Transparency Initiative
NGOs: Non-government organisations
