# Supplementary material for: A framework of NGO inside and outside strategies in the commercial determinants of health: findings from a narrative review
Source: Global Health. 2023 Oct 10;19:74. doi: 10.1186/s12992-023-00978-x (PMC10565967; doi:10.1186/s12992-023-00978-x)
Supplement: Supplementary file 1 — Additional file 1: Supplementary Table. Included studies. [file 12992_2023_978_MOESM1_ESM.docx]

**Supplementary Table: Included studies**

| **#** | **Author(s)** | **Title** | **Year** | **Methodology** | **Industry Sector** | **Time period of study** | **NGOs studied** | **NGO aims** | **Level of analysis / case country/ies** |
| --- | --- | --- | --- | --- | --- | --- | --- | --- | --- |
| **1** | Aaronson, S.L | Limited partnership: business, government, civil society, and the public in the extractive industries transparency initiative (EITI) | 2011 | Mixed | Extractive | 2008 | Global Witness, Catholic Agency for Overseas, Publish what you pay coalition. Transparency International, Revenue Watch Institute | Transparency of extractive industries | Global |
| **2** | Aguilar-Støen, M., Hirsch, C. | Bottom-up responses to environmental and social impact assessments: A case study from Guatemala | 2021 | Qualitative | Extractive | 2009 -2014 | La Puya Resistance, Oxfam, unnamed Guatemalan NGO, unnamed Norwegian NGO, Frente por la Defensa de San Miguel Ixtahuacan, the Mining Injustice Solidarity Network, and the Mesoamerican Movement against the extractive model. | Resistance against mining and hydropower developments | Guatemala |
| **3** | Ahaibwe, G., Abdool Karim, S., Thow, A.M., Erzse, A., Hofman, K. | Barriers to, and facilitators of, the adoption of a sugar sweetened beverage tax to prevent non-communicable diseases in Uganda: a policy landscape analysis | 2021 | Qualitative | Food | 2011 - 2021 | Uganda NCD Alliance, Tax Justice Alliance Uganda, SEATINI, Action Aid | Prevention, detection, management and reduction of NCDs | Uganda |
| **4** | Akin-Onitolo, A., Hawkins, B. | Framing tobacco control: the case of the Nigerian tobacco tax debates | 2022 | Qualitative | Tobacco | 2016 -2018 | Africa Tobacco Control Alliance, Campaign for Tobacco-free Kids, Civil Society Legislative Advocacy Centre, Environmental Rights Action / Friends of the Earth Nigeria and TC researchers represented the National Tobacco Control Alliance. | Advocate for higher taxes on tobacco products | Nigeria |
| **5** | Alexander, D., Tirin-Tudor, A., Dragu, I. | Implications of corporate accountability on civil society: The case of Rosia Montana Gold Corporation (RMGC) | 2018 | Qualitative | Extractive | 1997 -2015 | Alburnus Maior, Greenpeace, the International Council on Monuments and Sites, Mining Patrimonial Associations, and Patronal Confederation in Industry, Services and Trade. | Opponents (and supporters) of the Rosia Montana project | Rosia Montana, Apuseni Mountains, Romania |
| **6** | Alger, J., Dauvergne, P. | The Global Norm of Large Marine Protected Areas: Explaining variable adoption and implementation | 2017 | Qualitative | Food | 2006 -2016 | Pew Charitable Trusts, National Geographic Society, Conservation International, Australian Marine Conservation Society, the Marine Reserve Coalition in the UK, Ebiil Society | Marine conservation with specific focus on large scale protection of pelagic ecosystems | NW Pacific, USA; Australia; Palau, Pitcairn Islands, UK overseas territory |
| **7** | Alger, J., Dauvergne, P. | The Politics of Pacific Ocean Conservation: Lessons from the Pitcairn Islands Marine Reserve | 2017 | Qualitative | Food | 2015-2016 | Pew Charitable Trusts, National Geographic Society, Conservation International | Marine conservation through marine protection areas | Pitcairn Islands, (UK overseas territory) |
| **8** | Alper, D.K. | Transboundary environmental relations in British Columbia and the Pacific northwest | 1997 | Qualitative | Extractive | 1980 - 1995 | National Resources Defense Council, Greenpeace International, World Wildlife Fund, the Rainforest Action Network, Tatshenshini International, People for Puget Sound, Save Georgia Strait Alliance, Cascades International Alliance, Northwest Ecosystem Alliance. | Environmental protection for wilderness areas in the Pacific Northwest. | British Columbia and the Pacific Northwest (Canada and United States of America) |
| **9** | Anaf, J., Baum, F., Fisher, M., Friel, S. | Civil society action against transnational corporations: implications for health promotion | 2020 | Qualitative | Food, Extractive | N/A | Unnamed NGOs | To change transnational corporations practices related to health | Australia |
| **10** | Anderson, E., Zaloznaya, M. | Global civil society and the test of Kyoto: A theoretical extension | 2018 | Qualitative | Extractive | 2005-2012 | Australian Conservation Foundation, WWF, The Wilderness Society, Greenpeace, Friends of the Earth (Japan), Greenpeace (Japan), National Wildlife Federation, Conservation International, World Resources Institute, Worldwatch Institute, the Kiko Forum, David Suzuki Foundation, Environmental Defense Fund, Greenpeace Canada, Climate Action Network. | For governments to adopt and implement the Kyoto protocol | Japan, Canada, Australia |
| **11** | Andia, T., Chorev, N. | Making knowledge legitimate: transnational advocacy networks’ campaigns against tobacco,  infant formula and pharmaceuticals | 2017 | Qualitative | tobacco, food, pharmaceuticals | 1981 - 2003 | International Baby Food Action Network, Infant Feeding Action Coalition (subsequently known as Corporate Accountability International), Health Action International, National Interagency Council on Smoking and Health, multiple physicians associations, National Action on Smoking and Health, the Campaign for Tobacco-Free Kids, Framework Convention Alliance | Regulation of multinational companies to protect the health of vulnerable populations | Global |
| **12** | Anozie, M.C., Wingate, E.O. | NGO standing in petroleum pollution litigation in Nigeria - Centre for Oil Pollution Watch v Nigerian National Petroleum Corporation | 2020 | Qualitative | Extractive | 2003 - 2019 | Center for Oil Pollution Watch | Transparency and accountability of extractive industries | Nigeria |
| **13** | Anyidoho, N.A., Crawford, G. | Leveraging national and global links for local rights advocacy: WACAM's challenge to the power of transnational gold mining in Ghana | 2014 | Qualitative | Extractive | 1992 - 2013 | Wassa Association of Communities Affected by Mining | Advocates for the rights of local mining communities | Ghana |
| **14** | Apostol, O.M. | A project for Romania? The role of the civil society’s counter-accounts in facilitating  democratic change in society | 2015 | Qualitative | Extractive | 1997 - 2014 | Greenpeace (International) in collaboration with Romanian national NGOs and CSOs | Opposition to and mobilisation against exploitation of mineral resources, and development of a new mine. | Rosia Montania, Apuseni Mountains, Romania |
| **15** | Armstrong, C.W., van den Hove, S. | The formation of policy for protection of cold-water coral off the coast of Norway | 2008 | Qualitative | Food, Extractive | 1998 | WWF Norway, Bellona | Main focus is environmental advocacy | Norway |
| **16** | Arond, E., Bebbington, A., Luis Dammert, J. | NGOs as innovators in extractive industry governance. Insights from the EITI process in Colombia and Peru | 2019 | Qualitative | Extractive | 2013 -2018 | Puruvian NGOs that participated in the National Commission of EITI, Mesa de Sociedad Civil para la Transparencia en las Industrias Extractivas), Foro Nacional por Colombia, Fundación Avina | Engagement and participation in the Extractive Industries Transparency Initiative (EITI) process | Peru, Colombia |
| **17** | Arup, C, Dixon, J., Paul-Taylor, J. | The essential ingredients of food regulatory governance | 2020 | Qualitative | Food | 2011-2020 | Obesity Policy Coalition | Advocacy for initiatives to reduce obesity | Australia |
| **18** | Aslani, K. | Azerbaijan: Civil society and the petroleum sector | 2017 | Qualitative | Extractive | 2006 - 2016 | National Budget Group of Azerbaijan, Coalition for Transparency in Extractive Industries of Azerbaijan | Engagement and participation in the Extractive Industries Transparency Initiative (EITI) process | Azerbaijan |
| **19** | Aspinwall, M. | Bringing rights to life: How civil society organizations help guarantee participation rights in developing countries | 2021 | Qualitative | Extractive | 2003 - 2021 | PODER, IMDEC, CESDER, Río Sonora Basin Committees, Wirikuta Defense Front | Engagement and participation in environmental impact assessments (EIA) | Mexico |
| **20** | Aydin, C.I., Ozkaynak, B., Rodriguez-Labajos, B., Yenilmez, T. | Network effects in environmental justice  struggles: An investigation of conflicts between mining companies and civil society  organizations from a network perspective | 2017 | Mixed | Extractive | 2011-2015 | 1092 NGOs and supporting organisations | Mobilisation against mining | Global |
| **21** | Bainus, A., Yulianti, D. | Civil Society and the Struggle for Food sovereignty in Indonesia | 2016 | Qualitative | Food | 2004 - 2015 | Seknas Tani Jokowi, Serikat Petani Indonesia, Konsorsium Pembaruan Agraria-Agrarian Reform Consortium, Bina Desa Sadajiwa Foundation, Serikat Petani Indonesia-Indonesian farmers union, Koalisi Rakyat untuk Kedaulatan Pangan-peoples coalition for food sovereignty, Farmer Initiatives for Ecological Literacy and Democracy. | Advocacy for food sovereignty via agrarian reform. | Indonesia |
| **22** | Balbach, E.D., Glantz, S.A. | Tobacco control advocates must demand high-quality media campaigns: the California experience | 1998 | Qualitative | Tobacco | 1996 -1997 | American Cancer Society, American Heart Association, and Americans for Non-smokers’ Rights | Advocacy for tobacco control | California, USA. |
| **23** | Bandy, J. | Paradoxes of Transnational Civil Societies under Neoliberalism: The Coalition for Justice in the Maquiladoras | 2004 | Qualitative | General | 1996 -2002 | Coalition for Justice in the Maquiladoras | Opposition to labour and environmental injustices. | Mexico, USA, Canada |
| **24** | Barandiaran, J., Rubiano-Galvis, S | An empirical study of EIA litigation involving energy facilities in Chile and Colombia | 2019 | Mixed | Extractive | 1993 -2017 | Unnamed NGOs | Use of litigation invoking the rights to live in a clean environment and greater participation in decision-making. | Chile, Colombia |
| **25** | Barquera, S., Campos, I., Rivera, J.A. | Mexico attempts to tackle obesity: the process, results, push backs and future challenges | 2013 | Quantitative | Food | 2005 -2013 | Mexican Alliance for Healthy Nations, and other national and inter-American NGOs | Advocacy for prevention of obesity and support of the National Agreement for Healthy Nutrition. | Mexico |
| **26** | Barry, C.M, Clay, K.C., Flynn, M.E. | Avoiding the Spotlight: Human Rights Shaming and Foreign Direct Investment | 2013 | Quantitative | General | 1994 -2004 | Amnesty International, United Nations Commission on Human Rights, The Free-Burma Coalition, Sudan Divestment Taskforce | Advocacy for human rights | Global |
| **27** | Bassi, S., Ralston, R., Arora, M., Chugh, A., Nazar, G.P., Collin, J. | Understanding the dynamics of notification and implementation of Article 5.3 across India’s states and union territories | 2022 | Qualitative | Tobacco | 2015 -2019 | Unnamed NGOs | Advocacy for tobacco control policies | India |
| **28** | Basu, P. | Mining in Rajasthan—a Quagmire for Policy, Practice and People | 2020 | Qualitative | Extractive | 1994 - 2020 | Mine Labour Protection Campaign Trust, Rajasthan State Mine Labour Union | Collective action for workers’ rights and livelihoods | Rajasthan, North West India |
| **29** | Bebbington, A., Bebbington, D.H., Bury, J., Lingan, J., Munoz, J.A., | Mining and Social Movements: Struggles Over Livelihood and Rural Territorial Development in the Andes | 2007 | Qualitative | Extractive | 1990s -2000s | Defensa y Conservacion Ecologica de Intag, unnamed US NGOs, Acción Ecológica, Friends of the Earth International | Opposition to mine expansion and development, environmental education and awareness. | Peru, Ecuador |
| **30** | Berlie, L.S | Alliances for Sustainable Development: Business and NGO Partnerships | 2010 | Qualitative | Extractive | 1990s - 2020 | World Wildlife Fund International | To influence corporate practices to reduce environmental impact. | Global |
| **31** | Berridge, V., Hall, W., Taylor, S., Gartner, C., Morphett, K. | A first pass, using pre-history and contemporary history, at understanding why Australia and England have such different policies towards electronic nicotine delivery systems, 1970s–c. 2018 | 2021 | Qualitative | Tobacco | 1970s - 2018 | English ASH, New Nicotine Alliance, New Nicotine Alliance (Australia), An Australian Tobacco Harm Reduction Association, an Australian branch of ASH | Support (and opposition) to vaping, ENDs, NRT over time. | United Kingdom, Australia |
| **32** | Berteletti, F., King, J., Burch, J., Friedlaender, A.T., | Campaign for a revised Tobacco Products Directive in the European Union: lessons learnt | 2017 | Qualitative | Tobacco | 2009-2014 | Smoke Free Partnership | Advocacy for taxation and smoke free policies | European Union (EU) |
| **33** | Betsill, M. | Chapter 22: NGOs (Book Title: Research Handbook on Climate Governance) | 2015 | Qualitative | Extractive | 1980s- 2010s | Unnamed NGOs | Advocacy for climate change policy | Global |
| **34** | Bhatta, D.N., Crosbie, E., Bialous, S., Glantz, S. | Defending Comprehensive Tobacco Control Policy Implementation in Nepal From Tobacco Industry Interference (2011–2018) | 2020 | Qualitative | Tobacco | 2011-2019 | The Nepal Cancer Relief Society, Health and Environment Awareness Forum Nepal, Resource Centre for Primary Health Care, and Action Nepal, International Union against Tuberculosis and Lung Disease | Advocacy for tobacco control | Nepal |
| **35** | Bhatta, D.N., Bialous, S., Crosbie, E., Glantz, S. | Exceeding WHO Framework Convention on Tobacco Control (FCTC) Obligations: Nepal Overcoming Tobacco Industry Interference to Enact a Comprehensive Tobacco Control Policy | 2020 | Qualitative | Tobacco | 1986 - 2018 | National Health Education, Information and Communication Center, Nepal Cancer Relief Society, the Resource Center for Primary Health Care, Non-Smokers' Rights Association of Nepal, Health and Environment Awareness Forum Nepal | Advocacy for tobacco control and legislation. | Nepal |
| **36** | Bhatta, D., Crosbie, E., Bialous, S., Glantz, S. | Tobacco control in Nepal during a time of government turmoil (1960–2006) | 2020 | Qualitative | Tobacco | 1950-2006 | Mrigendra Samjhana Medical Trust, Nepal Medical Association, Nepal Cancer Relief Society, PROPUBLIC, Resource Center for Primary Health Care, Non-Smokers’ Rights Association of Nepal | Advocacy for tobacco control | Nepal |
| **37** | Bicakci, A.B | Public Relations towards Member Engagement in Advocacy Networks: the 'No Pesticides on My Plate' Campaign | 2021 | Qualitative | Food | 2019 | Advocacy network, led by Bugday Association | Advocacy for alternative farming and pest management methods | Turkey |
| **38** | Bieri, F. | From blood diamonds to the Kimberley Process: How NGOs cleaned up the global diamond industry | 2016 | Qualitative | Extractive | 1999 - 2009 | Partnership Africa Canada, Global Witness, Amnesty International, Fatal Transactions, Oxfam | To raise awareness on the issue of conflict diamonds | Global |
| **39** | Bloomfield, M.J. | Shame campaigns and environmental justice: corporate shaming as activist strategy | 2014 | Qualitative | Extractive | 2000 - 2010 | The No Dirty Gold (NDG) campaign (Earthworks and Oxfam)  The Global Finance Campaign (Rainforest Action Network) | To change industry practices by targeting the reputational value of individual firms. | Global |
| **40** | Bostick, K. | NGO approaches to minimizing the impacts of aquaculture: A review | 2008 | Qualitative | Food | 1970-2005 | Fundacion Terram (Chile), Oxfam, WWF-US, WWF-Norway, Monteray Bay Aquarium (US), Marine Conservation Society (UK), North Sea Foundation (Netherlands), Fundacion Huinay (Chile), IUCN, Environmental Defense, Canadian Alliance for Aquaculture Reform | Advocacy on a range of issues related to aquaculture | Canada, UK, Norway, Chile, Netherlands, US |
| **41** | Bowles, P., MacPhail, F. | The town that said "No" to the Enbridge Northern Gateway pipeline: The Kitimat plebiscite of 2014 | 2017 | Qualitative | Extractive | 2012 - 2014 | Douglas Channel Watch | Opposition to the development of the Enbridge Northern Gateway pipeline | Canada (British Columbia) |
| **42** | Calderon, B.C., Naef, J., Tokarski, K.O. | Multinational corporations and social responsibility | 2016 | Qualitative | General | 2012 | Unnamed NGOs involved in the Swiss Campaign for Corporate Justice. | Advocacy for social issues and environmental issues. | Switzerland |
| **43** | Calhoun, D. | Decreasing the Supply of and Demand for Guns: Oakland's Youth Advocacy Project | 2014 | Qualitative | Weapons | 1988 -2004 | Youth ALIVE! | Preventing youth gun violence through peer education and a leadership program | California (USA) |
| **44** | Carriedo, A., Koon, A.D., Encarnacion, L.M., Lee, K., Smith, R., Walls, H. | The political economy of sugar-sweetened beverage taxation in Latin America: lessons from Mexico, Chile and Colombia | 2021 | Qualitative | Food | 2011-2018 | Allianza por la Salud Alimentaria, El Poder del Consumidor, Fundacion Midete, Contrapeso, Bloomberg Philanthropies, Frente por un Chile Saludable, Alianza por la Salud Alimentaria, Educar Consumidores. | Advocacy for the introduction of sugar sweetened beverage taxation | Mexico, Chile, Colombia |
| **45** | Carriedo, A, Lock, K., Hawkins, B. | Policy Process And Non-State Actors' Influence On The 2014 Mexican Soda Tax | 2020 | Qualitative | Food | 2014 | International organisations, national think tanks and civil society organisations. | Support for a soda tax and promotion of evidence of health effects | Mexico |
| **46** | Castello, I., Lopez-Berzosa, D. | Affects in Online Stakeholder Engagement: A Dissensus Perspective | 2021 | Mixed | General | 2008 - 2020 | Plastic Pollution Coalition, Algalita and 5 Gyres | Reduction of plastic production and consumption | Global |
| **47** | Cezne, E. | Forging transnational ties from below: Challenging the Brazilian mining giant Vale S.A. across the South Atlantic | 2019 | Qualitative | Extractive | 2018 - 2019 | International Articulation of those Affected by Vale. | Contestation of Vale’s corporate practices and mining activities worldwide | Brazil, Mozambique, Canada |
| **48** | Champagne, B.M, Sebrie, E., Schoj, V. | The role of organized civil society in tobacco control in Latin America and the Caribbean | 2010 | Qualitative | Tobacco | 1970s-2009 | A coalition of Latin American, Caribbean, Canadian and USA NGOs. | Tobacco control in Latin America and the Caribbean. | regional: Latin America; Caribbean |
| **49** | Chapman, J., Fisher, T. | The effectiveness of NGO campaigning: Lessons from practice | 2000 | Qualitative | Food, Textile | 1997-1998 | Ghanaian Infant Nutrition Action Network, unnamed Indian and German NGOs | Promotion of breastfeeding in Ghana; Advocacy against the use of child labour in the carpet industry in India | Ghana; Uttar Pradesh, India |
| **50** | Colli, F., | Politics without bureaucrats? Western European NGO campaigns in the state and the market | 2021 | Mixed | Food, and General | 2018 | The study focuses on 24 NGO campaigns (unnamed NGOs) across four issues (cage eggs, antibiotic use in farming, ocean plastics and civil rights and technology) | Campaigns against state, market or both on a range of issues | UK and Italy |
| **51** | Coumans, C. | Realising solidarity: Indigenous peoples and NGOs in the contested terrains of mining and corporate accountability | 2017 | Qualitative | Extractive | 1996 - 2007 | Earthworks, Oxfam Australia, Oxfam America, unnamed Peruvian NGOs, Rhéébù Nùù, MiningWatch Canada, unnamed from international and Philippines NGOs, Kairos, Development and Peace, Christian Aid, Rights and Democracy, Western Mining Action Network, the Ontario Mining Action Network, Mines and Communities, London Mining Network< CAFOD, The North-South Institute | Advocacy for indigenous rights, authority and protection of lands from development of mining projects. | USA, Peru,  New Caledonia, Philippines |
| **52** | Crosbie, E., Sosa, P., Glantz, S.A. | Defending strong tobacco packaging and labelling regulations in Uruguay: transnational tobacco control network versus Philip Morris International | 2018 | Qualitative | Tobacco | 2014-2016 | Uruguayan CSOs in collaboration with US-based Campaign for Tobacco Free Kids, supported by the Bloomberg Initiative to Reduce Tobacco Use and the Framework Convention Alliance. | Advocacy for tobacco control | Uruguay |
| **53** | Crosby, A., Dunn, J.L., Aditjondro, E., Rachfiansyah | Tobacco Control Is a Wicked Problem: Situating Design Responses in Yogyakarta and Banjarmasin | 2019 | Qualitative | Tobacco | 2017 - 2018 | Vital Strategies | Advocate for equitable and affective public health systems | Indonesia |
| **54** | Debrah, E., Graham, E. | Preventing the oil curse situation in Ghana: The role of civil society organisations | 2015 | Qualitative | Extractive | 2013 | Civil Society Platform on Oil and Gas (coalition) | Enhance transparency and accountability in the production and management of oil wealth in Ghana. | Ghana |
| **55** | Delina, L.L. | Coal development and its discontents: Modes, strategies, and tactics of a localized, yet networked, anti-coal mobilisation in central Philippines | 2022 | Qualitative | Extractive | 2013-2018 | The Bohol anti-coal movement | Opposed to development of a proposed coal-fired power plant | (Visayas Islands, Boho province) Central Philippines |
| **56** | Denedo, M., Thomson, I., Yonekura, A. | International advocacy NGOs, counter accounting, accountability and engagement | 2017 | Qualitative | Extractive | 2015 | International, regional, national, local NGOs and civil society. | To improve governance and accountability in relation to human rights and environmental protection. | Nigeria (Niger Delta) |
| **57** | Denedo, M., Thomson, I., Yonekura, A. | Accountability, maps and inter-generational equity: evaluating the Nigerian oil spill monitor | 2018 | Qualitative | Extractive | N/A | The Nigerian Oil Spill Monitor | To audit, verify and challenge official accounts of oil corporations following oil spills. | Nigeria (Niger Delta) |
| **58** | Denedo, M., Thomson, I., Yonekura, A. | Ecological damage, human rights and oil: local advocacy NGOs dialogic action and alternative accounting practices | 2019 | Qualitative | Extractive | 2015 | Unnamed NGOs | To improve transparency and to hold public officials accountable to the people in the Niger Delta. | Nigeria (Niger Delta) |
| **59** | Drope, J. | The politics of smoke-free policies in developing countries: Lessons from Africa | 2010 | Qualitative | Tobacco | 2009 -2010 | Association Burkinabe de Sante´ Publique, Canadian Public Health Association, Union des associations contre le tabac, Tobacco-Free Schools Environment Initiative, Institute for Legislative Affairs, ViSa, Nigerian Heart Foundation and Environmental Right Action, Friends of the Earth Nigeria, South Africa’s Tobacco Action Group, National Council Against Smoking, Cancer Association of South Africa, Heart and Stroke Foundation South Africa, Tanzania Public Health Association, Zambia Tobacco Control Consortium. | Advocacy for tobacco control and implementation of smoke-free policies | Burkina Faso, Cameroon, Eritrea, Ghana, Kenya, Malawi, Mauritius, Nigeria, Senegal, South Africa, Tanzania, Zambia |
| **60** | Dry, T., Baker, P. | Generating Political Commitment for Regulatory Interventions Targeting Dietary Harms and Poor Nutrition: A Case Study on Sugar-Sweetened Beverage Taxation in Australia | 2021 | Qualitative | Food | 2010 - 2020 | Public Health Association of Australia, CHOICE, Australian Medical Association, Obesity Policy Coalition, Sugar By Half, That Sugar Movement, Sugar Free Smiles, Parents’ Voice, YMCA, Consumers Health Forum, CHOICE, Queensland Country Women’s Association, and Australian Council of Social Service. | Support for a sugar sweetened beverage tax | Australia |
| **61** | Edge, S., Eyles, J. | Contested Governmentalities: NGO enrolment and influence over chemical risk governance rationales and practices | 2015 | Qualitative | General | 1999 -2014 | Canadian Environmental Network | To influence stronger chemical governance in Canada | Canada |
| **62** | Farraiolo, K. | Messaging and Advocacy in U.S. Tobacco Control Policy, 2009–19 | 2019 | Qualitative | Tobacco | 2009 -2019 | The Truth Initiative and the Campaign for Tobacco‐Free Kids | Advocacy for tobacco control | United States of America |
| **63** | Filer, C., Mahanty, S., Potter, L. | The FPIC Principle Meets Land Struggles in Cambodia, Indonesia and Papua New Guinea | 2020 | Qualitative | Extractive | N/A | International Federation for Human Rights, Worldwide Fund for Nature, The Alliance of Indigenous People of the Archipelago, Sawit Watch, Forest Peoples Programme, The Forest Trust, The Rainforest Alliance | Advocacy for local communities affected by a particular project or investment, asserting centrality of free prior informed consent (FPIC). | Cambodia, Indonesia, Papua New Guinea |
| **64** | Fisher, D.R., Galli, A.M. | Civil society | 2015 | Qualitative | Extractive, General | 2009 -2014 | Climate Action Network, Climate Justice Now!, Climate Justice Action | Advocacy for stronger government action on climate change | Global |
| **65** | Ford, N. | Patents, access to medicines and the role of non-governmental organisations | 2004 | Qualitative | Pharmaceuticals | 1998 - 2004 | Medecins Sans Frontieres, Consumer Project on Technology, Act-UP and Health Gap, Oxfam, Voluntary Service Overseas (VSO) and Action Aid, Treatment Action Campaign in South Africa and the Aids Access Coalition in Thailand | Advocacy for regulation for improved access to affordable medicines | South Africa, Kenya, Guatemala, Thailand, global |
| **66** | Friel, S., Baker, P., Thow, A., Gleeson, D., Townsend, B., Schram, A. | An exposé of the realpolitik of trade negotiations: Implications for population nutrition | 2019 | Qualitative | Food | 2016 | Unnamed NGOs | To influence trade agreement decision-making processes in favour of population nutrition. | Australia |
| **67** | Geidne, S., Eriksson, C. | Working with or against the stores?: A non-governmental organization working with alcohol purchase attempts | 2009 | Mixed | Alcohol | 2003 - 2006 | Swedish Temperance Movement (UNF) | To reduce the rate of successful purchase attempts of medium-strength beer in Sweden | Sweden |
| **68** | Gneiting, U. | From global agenda-setting to domestic implementation: successes and challenges of the global health network on tobacco control | 2016 | Qualitative | Tobacco | 2012 | Action on Smoking and Health, Framework Convention Alliance, the Campaign for Tobacco-Free Kids, the Southeast Asia Tobacco Control Alliance, the African Tobacco Control Alliance, and some national level tobacco control groups. | Advocacy for tobacco control and FCTC implementation | Global |
| **69** | Guay, T., Doh, J.P., Sonclair, G. | Non-governmental organizations, shareholder activism, and socially responsible investments: Ethical, strategic, and governance implications | 2004 | Qualitative | General | N/A | Humane Society of the United States, the Sierra Club, Friends of the Earth (FOE), Shareholder Action Network, ICCR, Institutional Investor Responsibility Center, CERES | To influence investors on social and environmental causes | Global |
| **70** | Hall, N.L., Taplin, R. | Solar festivals and climate bills: Comparing NGO climate change campaigns in the UK and Australia | 2007 | Qualitative | Extractive | 2004 -2006 | Stop Climate Chaos (the umbrella network of UK NGOs), Climate Action Network Australia (Australian branch of an international collaboration of NGOs focused on climate change) | To influence political action on climate change and reduce reliance on extractive industries. | UK, Australia |
| **71** | Hamman, E | The influence of environmental NGOs on project finance: a case study of activism, development and Australia’s Great Barrier Reef | 2016 | Qualitative | Extractive | 2015 - 2016 | Collevecchio Declaration, BankTrack, BankWatch, Netwerk Vlaanderen and the Berne Declaration, Friends of the Earth France, Attac France and Bizi! | To exert influence on the finance industry not to finance extractive projects | Australia, France |
| **72** | Harris, J., Frongillo, E.A., Nguyen, P.H., Kim, S.S., Menon, P. | Changes in the policy environment for infant and young child feeding in Vietnam, Bangladesh, and Ethiopia, and the role of targeted advocacy | 2017 | Qualitative | Food | 2010 -2014 | Alive & Thrive | To improve policy for infant and young child feeding (IYCF) and child undernutrition. | Vietnam, Bangladesh, Ethiopia |
| **73** | Haslam, P.A., Godfried, J. | Activists and regulatory politics: Institutional opportunities, information, and the activation of environmental regulation | 2020 | Qualitative | Extractive | 2016-2018 | Union of Citizen Assemblies, Conciencia Solidaria, Greenpeace, AJNST, Observatorio latinoamericano de conflictos ambientales, Madres Jachalleras | Improved regulation of extractive industries. | Argentina, Chile |
| **74** | Holden, W.N. | Civil Society Opposition to Nonferrous Metals Mining in the Philippines | 2005 | Qualitative | Extractive | 2004 - 2005 | People’s organization Katawhang Simbahan Alangsa Malamboong Kabuhatan, Anislagan Bantay Kalikasan Task Force, Environmental Legal Assistance Center, National Council of Churches, Green Forum. | Advocacy and mobilisation against hardrock mining, in general, and against the Mining Act of 1995 in particular. | Philippines |
| **75** | Ivanova, M. | Shareholder activism and the ethical harnessing of institutional investors: The unique case of ShareAction | 2016 | Qualitative | Extractive | 2005 - 2012 | ShareAction | To influence shareholders to oppose extractive projects | UK, Canada |
| **76** | Jalbert, K., Malone Rubright, S., Edelstein, K. | The Civic Informatics of FracTracker Alliance: Working with Communities to Understand the Unconventional Oil and Gas Industry | 2017 | Qualitative | Extractive | 2010 - 2015 | FracTracker Alliance | To advance local moratoria and gas bans. | USA |
| **77** | Kashiwabara, M., Arul, R., Goswami, H., Narain, J.P., Armada, F. | Local governments and civil society lead breakthrough for tobacco control: lessons from Chandigarh and Chennai | 2011 | Qualitative | Tobacco | 2003 - 2009 | Tamil Nadu Tobacco Control Coalition, Smoke-Free Chennai Coalition, Burning Brain Society | Tobacco control | India |
| **78** | Lansang, L.G.F. | NGOs, coalition building and the campaign for a minerals management policy in the Philippines | 2011 | Qualitative | Extractive | 1990 - 1998 | Kalikasan People’s Network for the Environment, Defend Patrimony! Alliance, Alyansa Tigil Mina, the Legal Rights and Natural Resources Center, Inc. Kasama sa Kalikasan/Friends of the Earth Philippines, Alyansa Tigil Mina coalition, Alliance for Genuine Development, Tampakan People’s Crusade for Environmental Protection | Advocacy for an alternative mining bill | Philippines |
| **79** | Lencucha, R., Kothari, A., Labonte, R. | The role of non-governmental organizations in global health diplomacy: negotiating the Framework Convention on Tobacco Control | 2011 | Qualitative | Tobacco | 1999-2003 | Unnamed Canadian NGOs | Advocacy for a strong FCTC | Global |
| **80** | Lencucha, R., Labonte, R., Rouse, M.J. | Beyond idealism and realism: Canadian NGO/government relations during the negotiation of the FCTC | 2010 | Qualitative | Tobacco | 1999-2003 | Unnamed Canadian NGOs | Advocacy for a strong FCTC | Global |
| **81** | Lencucha, R., Ruckert, A., Labonte, R., Drope, J. | Opening windows and closing gaps: a case analysis of Canada's 2009 tobacco additives ban and its policy lessons | 2018 | Qualitative | Tobacco | 2008-2009 | Physicians for a Smoke Free Canada, Canadian Cancer Society, Quebec Coalition for Tobacco Control | Advocacy for tobacco control | Canada |
| **82** | Levkoe, C.Z., Sheedy, A. | A people-centred approach to food policy making: Lessons from Canada’s People’s Food Policy project | 2019 | Qualitative | Food | 2009 - 2011 | People’s Food Policy project / People’s Food Commission | Advocacy for transformation of the dominant food system | Canada |
| **83** | Lipschutz, R.D. | Sweating It Out: NGO Campaigns and Trade Union Empowerment | 2004 | Qualitative | Textile | 2001 | the 'clean clothes' movement | Stronger regulation of the textile industry | China, Indonesia, Philippines, Thailand, USA |
| **84** | Llewellyn, L. | Ecological Conflicts, Resistance, Leadership and Collective Action for Just Resilience: What Can We Learn from a Community Struggle Against a Proposed Coal Mine in Fuleni, KwaZulu-Natal, South Africa? | 2021 | Qualitative | Extractive | 2016 - 2017 | Mining Affected Communities United in Action | Opposition to a mining proposal in rural Fuleni, KwaZulu-Natal | Fuleni, Saint Lucia (KwaZulu-Natal, South Africa) |
| **85** | Lock, I., Statchel, C., Seele, P. | Traveling frames: How corporate and civil society actors try to influence public administration and courts in a case on nuclear emission data in Switzerland | 2019 | Qualitative | Extractive | 2013 - 2016 | Greenpeace Switzerland | Transparency on nuclear emissions data | Switzerland |
| **86** | Lysack, M. | Effective policy influencing and environmental advocacy: Health, climate change, and phasing out coal | 2015 | Qualitative | Extractive | 2008 - 2013 | Asthma Society of Canada, Lung Association of Alberta & Northwest Territories, and Canadian Association of Physicians for the Environment | Advocacy for a phase out of coal | Alberta, Canada |
| **87** | MacKay, B., Munro, I. | Information Warfare and New Organizational Landscapes: An Inquiry into the ExxonMobil-Greenpeace Dispute over Climate Change | 2021 | Qualitative | Extractive | N/A | Greenpeace | Stope ExxonMobil | Global |
| **88** | Malinowska-Sepruch, K., Bonnell, R., Hoover, J. | Civil society - a leader in HIV prevention and tobacco control | 2006 | Qualitative | Tobacco | 1990 - 2006 | Ukrainian Harm Reduction Association, Framework Convention Alliance, Health Promotion Foundation, For a Smoke-Free Kazakhstan | To enhance collaboration in pushing the government to adopt tobacco control legislation and health protection policies. | Poland, Kazakhstan, Ukraine |
| **89** | Mamudu, H.M., Glantz, S.A. | Civil society and the negotiation of the Framework Convention on Tobacco Control | 2009 | Qualitative | Tobacco | 2004 - 2007 | Framework Convention Alliance | Advocate for strong and effective tobacco control legislation | Global |
| **90** | Martinez, H., Pederson, A. | Global frameworks, local strategies: Women's rights, health, and the tobacco control movement in Argentina | 2019 | Qualitative | Tobacco | N/A | Argentine Smoke-Free Alliance, Inter American Heart Foundation, Fundación Interamericana del Corazón Argentina | Advocate for strong and effective tobacco control legislation | Argentina |
| **91** | Matthes, B.K., Robertson, L., Gilmore, A.B. | Needs of LMIC-based tobacco control advocates to counter tobacco industry policy interference: insights from semi-structured interviews | 2020 | Qualitative | Tobacco | 2019 | Unnamed NGOs | Advocacy on tobacco control and other public health issues | Bangladesh, India, Sri Lanka, Ethiopia, Uganda, Zambia, Colombia, Ukraine |
| **92** | Mbatu, R.S., | Discourses of FLEGT and REDD + regimes in cameroon: A nongovernmental organization and international development agency perspectives | 2020 | Qualitative | Extractive | 2014 - 2016 | World Wildlife Fund for Nature, Center for Environment and Development, International Union for Conservation of Nature, Cameroon Living Earth Foundation, Wildlife Conservation Society, International Center for the Support of Sustainable Development, Centre for International Development and Training | To exert influence on the FLEGT and REDD+ regimes within Cameroon | Cameroon |
| **93** | Mbariza, C., Namatovu, T. | Civic space and human rights advocacy in the extractive industry in Uganda: Implications of the 2016 Non-Governmental Organisations Act for oil and gas civil society organisations | 2018 | Qualitative | Extractive | 2006 - 2016 | Civil Society Coalition for Oil, Oil Change International, Publish What You Pay, Civicus, Eastern and Horn of Africa Human Rights Defenders Project, Action Aid, Global Rights Alert. | Transparency and accountability in the management of revenue in the oil and gas sector | Uganda |
| **94** | McCool, J., McKenzie, J., Lyman, A., Allen, M., | Supporting Pacific Island Countries to Strengthen Their Resistance to Tobacco Industry Interference in Tobacco Control: A Case Study of Papua New Guinea and Solomon Islands | 2013 | Qualitative | Tobacco | 2012 - 2013 | Bloomberg Project team, Tobacco Control Task Force (Solomon Islands), University of PNG (PNG) | Advocate for strong and effective tobacco control legislation | Papua New Guinea, Solomon Islands |
| **95** | McDaniel, P., Malone, R., | British American Tobacco's partnership with Earthwatch Europe and its implications for public health | 2012 | Qualitative | Tobacco | 1997 - 2009 | Earthwatch Europe | Partnership with corporation in aid of environmental protection and conservation | Global |
| **96** | McDonald, D., Stirling, R. | Evaluating the capacity building roles of the state and territory peak bodies in the Australian alcohol and other drug sector | 2019 | Qualitative | Alcohol | 2012 - 2014 | Peaks Capacity Building Network | Seek to reduce alcohol and drugs related harms | Australia |
| **97** | McKenzie, J., Carter, A.V., | Stepping stones to keep fossil fuels in the ground: Insights for a global wind down from Ireland | 2021 | Qualitative | Extractive | 2019 | Trócaire, Stop Climate Chaos Coalition, Friends of the Earth, Extinction Rebellion, and Not Here Not Anywhere, 350.org, Global Catholic Climate Movement and Green Faith | Advocacy for keeping fossil fuels in the ground / banning fossil fuel production. | Ireland |
| **98** | Murombo, T. | The Effectiveness of Initiatives to Promote Good Governance, Accountability and Transparency in the Extractives Sector in Zimbabwe | 2016 | Qualitative | Extractive | 2000 - 2013 | The Africa Initiative on Mining, Environment and Society, Publish What You Pay, Kimberly Process Certification Scheme and Civil Society Coalition | Transparency and accountability in the natural resources sphere of the economy | Zimbabwe |
| **99** | Murphy-Gregory, H., Elbra, A., Mikler, J., Johnson, L. | The Australian campaign against corporate tax avoidance: agenda-setting, narratives, and political opportunities | 2020 | Qualitative | General | 2017 | Tax Justice Network | Campaign on corporate tax avoidance and advocate for tax justice | Australia |
| **100** | Mzembe, A.N., Meaton, J. | Driving Corporate Social Responsibility in the Malawian Mining Industry: A Stakeholder Perspective | 2014 | Qualitative | Extractive | 2009 -2010 | Friends of the Earth International, CHRR, KADET, FOCUS and CCJP | Opposition to development of a uranium mine | Malawi |
| **101** | Nakkash, R.T., Torossian, L., Hajj, T.E., Khalil, J., Afifi, R.A. | The passage of tobacco control law 174 in Lebanon: reflections on the problem, policies and politics | 2018 | Qualitative | Tobacco | 2009 - 2011 | American University of Beirut Tobacco Control Research Group, TFI, Indyact | Prevention and control of tobacco use and its consequences | Lebanon |
| **102** | Neville, K.J., Weinthall, E. | Scaling up site disputes: strategies to redefine 'local' in the fight against fracking | 2018 | Qualitative | Extractive | 2013 - 2015 | Yukoners Concerned, Frack-Free Yukon, Yukon Conservation Society, and the Yukon chapter of the Canadian Parks and Wilderness Society | Opposed expansion of LNG and fracking industry activities in the Yukon | Yukon province, Canada |
| **103** | North, L.L., Young, L. | Generating rights for communities harmed by mining: legal and other action | 2013 | Qualitative | Extractive | 1995 - 2011 | Recherches Internationales Quebec, Canadian Association against Impunity (comprised of unnamed NGOs from the Congo, the United Kingdom and Canada), a group of nine Canadian civic and union organisations, Observatory of Indigenous Peoples’ Rights | Advocacy for human rights and litigation against Canadian mining corporations on behalf of affected communities | Mexico, DRC, Ecuador, Guyana, Canada |
| **104** | Obi, C. | Nigeria: The role of civil society in the politics of oil governance and revenue management | 2017 | Qualitative | Extractive | 1990s - 2015 | The Nigerian Labour Congress, the Trade Union Congress, the Petroleum and Natural Gas Senior Staff Association of Nigeria, the National Union of Petroleum and Natural Gas Workers, Revenue Watch Institute, the Nigerian Extractive Industries Transparency Initiative, Publish What You Pay Nigeria, BudgiT, the Oil Revenue Tracking Initiative, Environmental Rights Action, the Stakeholder Democracy Network, the Civil Liberties Organization, Social Action, the Ijaw Youth Council, the Movement for the Survival of the Ogoni People, Civil Society Legislative Advocacy Centre, the Centre for Leadership, Strategy and Development, and the Occupy Nigeria movement. | Seek to influence oil policy in Nigeria | Nigeria |
| **105** | Olesen, T. | 'In the court of public opinion': Transnational problem construction in the HIV/AIDS medicine access campaign, 1998-2001 | 2006 | Qualitative | Pharmaceuticals | 1998 - 2001 | OXFAM, Médecins Sans Frontières, Canadian HIV/AIDS Legal Network, Treatment Action Campaign, Consumer Project on Technology, Health GAP | Advocacy for access to HIV/AIDS medicine | Global, South Africa |
| **106** | Ollis, T. | Adult learning, circumstantial activism and ecological habitus in the coal seam gas protests | 2021 | Qualitative | Extractive | N/A | Friends of the Earth, Lock the Gate Alliance | To prevent multinational mining companies from ‘fracking’ for coal seam gas | Australia |
| **107** | Ollis, T., Hamel-Green, M. | Adult education and radical habitus in an environmental campaign: Learning in the coal seam gas protests in Australia | 2015 | Qualitative | Extractive | 2013 - 2014 | Friends of the Earth, Lock the Gate Alliance | To prevent multinational mining companies from ‘fracking’ for coal seam gas | Gippsland, Central Victoria, Australia |
| **108** | Ozanne, L.K., Smith, P.M. | Strategies and perspectives of influential environmental organizations toward tropical deforestation | 1993 | Qualitative | Extractive | 1987 - 1992 | A coalition of NGOs from West Germany, USA, Netherlands, Switzerland, Japan, Australia, Canada, UK, and Denmark. | To oppose extractive logging | Global |
| **109** | Paukšte, E., Liutkute, V., Štelemekas, M., Goštautaite Midttun, N., Veryga, A. | Overturn of the proposed alcohol advertising ban in Lithuania | 2014 | Qualitative | Alcohol | 2007 - 2011 | National tobacco and alcohol control coalition | Advocacy for an alcohol advertising ban | Lithuania |
| **110** | Phillips, R. | Is corporate engagement an advocacy strategy for NGOs?: The community aid abroad experience | 2002 | Qualitative | Extractive | 1999 - 2000 | Community Aid Abroad | Seeks to improve conditions for peoples affected by Australian-based mining companies operating in countries in the Asia-Pacific region. | Indonesia, Papua New Guinea, and the Philippines |
| **111** | Pieck, S.K, Moog, S.A. | Competing entanglements in the struggle to save the Amazon: The shifting terrain of transnational civil society | 2009 | Qualitative | Extractive | 2003- 2006 | Amazon Alliance, the Coordinating Body of Indigenous Organisations of the Amazon Basin | Advocacy for indigenous peoples’ rights | Global |
| **112** | Pillay, K., Maharaj, M. | The business of advocacy: A case study of Greenpeace | 2016 | Qualitative | Extractive | 2006 - 2011 | Greenpeace | Advocacy for environmental protection | Global,  Argentina |
| **113** | Price, R. | Reversing the gun sights: Transnational civil society targets land mines | 1998 | Qualitative | Weapons | N/A | International Campaign to Ban Landmines | Advocacy for an anti-personnel land mine ban | Global |
| **114** | Raitio, K., Saarikoski, H. | Governing Old-Growth Forests: The Interdependence of Actors in Great Bear Rainforest in British Columbia | 2012 | Qualitative | Extractive | 2009 | Multiple environmental non-government organisations and First Nations groups | Advocacy for environmental protection, opposition to logging | British Columbia, Canada |
| **115** | Rao Seshadri, S., Kaulgud, R., Jha, P. | 'You cannot touch taxes easily': Making the case for tobacco taxation in India | 2021 | Qualitative | Tobacco | N/A | Cancer Patients Aid Association | Tobacco control and implementation of the FCTC. | Karnataka, (India) |
| **116** | Rosser, A. | Contesting tobacco-control policy in Indonesia | 2015 | Qualitative | Tobacco | N/A | Indonesian Heart Foundation, Indonesian Cancer Foundation, Indonesian Women Against Tobacco, Indonesian Consumers Foundation, the Jakarta Citizens’ Forum, the Institute for Preventing Smoking Problems, Indonesia Corruption Watch, and the National Commission for Child Protection | Advocacy for tobacco control and development of tobacco control policies. | Indonesia |
| **117** | Roy Chowdury, A., Lahiri-Dutt, K. | Extractive capital and multi-scalar environmental politics: interpreting the exit of Rio Tinto from the diamond fields of Central India | 2021 | Qualitative | Extractive | 2017 | Coalition of national and international civil society groups | Conservation and environmental protection | India (Chattarpur, Bundelkhand region of Madhya Pradesh, central India) |
| **118** | Ruysschaert, D., Salles, D. | The strategies and effectiveness of conservation ngos in the global voluntary standards: The case of the roundtable on sustainable palm-oil | 2016 | Qualitative | Food  General | 2011 - 2014 | Conservation NGOs engaged in the Roundtable on Sustainable Palm Oil (RSPO) | To strengthen biodiversity conservation | South East Asia |
| **119** | Sabi, S.C., Rieker, M. | The role of civil society in health policy making in South Africa: a review of the strategies adopted by the Treatment Action Campaign | 2017 | Qualitative | Pharmaceuticals | 1998 - | Treatment Action Campaign (TAC) | Advocated for equitable treatment access for all people living with HIV/ AIDS in South Africa | South Africa |
| **120** | Schmitz, H.P. | The global health network on alcohol control: Successes and limits of evidence-based advocacy | 2016 | Qualitative | Alcohol | 1990s - 2010's | Global Alcohol Policy Alliance (GAPA) | Advocacy for global public health measures to reduce alcohol harm | Global |
| **121** | Selamoğlu, M., Fawkes, S., Önal, A.E., Gleeson, D. | Two steps forward, one step back: the lead up to tobacco plain packaging policy in Turkey | 2022 | Qualitative | Tobacco | 2018 | The National Coalition on Tobacco or Health (SSUK) | Advocacy and support for tobacco control and development of tobacco control policies. | Turkey |
| **122** | Simpson, A., Smits, M. | Transitions to Energy and Climate Security in Southeast Asia? Civil Society Encounters with Illiberalism in Thailand and Myanmar | 2018 | Qualitative | Extractive | 1988 - 2018 | Renewable Energy Association of Myanmar, and the Thailand Climate Justice Working Group | To critically address energy and climate issues | Thailand, Myanmar |
| **123** | Syarifuddin, K.A., Cangara, A.R., Rahman, I., Baharuddin, A., Apriliani, A. | The market campaign strategy of Greenpeace in decreasing rainforest deforestation in Indonesia: a case study of the usage of palm oil in Nestlé’s products | 2020 | Qualitative | Food | 2010 | Greenpeace | Advocacy on the causes of deforestation | Indonesia |
| **124** | Symons, K. | Transnational spaces, hybrid governance and civil society contestation in Mozambique’s gas boom | 2016 | Qualitative | Extractive | 2013 - 2014 | Civil Society Platform for Extractive Industries and Natural Resources (comprises around 20 organisations including Action Aid, Public Integrity Centre, Centro Terra Viva, Women, Gender and Development, and WWF Mozambique). | Advocacy for greater transparency and rights-based engagement | Mozambique |
| **125** | Ternes, B., Ordner, J., Cooper, D.H. | Grassroots resistance to energy project encroachment: Analyzing environmental mobilization against the Keystone XL Pipeline | 2020 | Qualitative | Extractive | 2011 - 2015 | Bold Nebraska, Bold Alliance | Opposition to the pipeline in Nebraska and the transnational KXL project | USA, Canada |
| **126** | Trevena, H., Petersen, K., Thow, A.M., Dunford, E.K., Wu, J.H.Y., Neal, B. | Effects of an advocacy trial on food industry salt reduction efforts—An interim process evaluation | 2017 | Mixed | Food | 2013 - 2015 | Unnamed NGOs | Reduction of salt content in processed foods | Australia |
| **127** | Tysiachniouk, M.S., Horowitz, L.S., Korkina, V.V., Petrov, A.N. | Indigenous-led grassroots engagements with oil pipelines in the U.S. and Russia: the NoDAPL and Komi movements | 2021 | Qualitative | Extractive | 2015 - 2020 | No Dakota Access Pipeline movement, the Save Pechora Committee | Opposed construction of oil pipelines and advocated on issues common to Indigenous Peoples’ struggles | USA, Russia |
| **128** | Uang, R., Crosbie, E., Glantz, S.A. | Tobacco control law implementation in a middle-income country: Transnational tobacco control network overcoming tobacco industry opposition in Colombia | 2018 | Qualitative | Tobacco | 2014 -2015 | Corporate Accountability International, Fundación para la Educación y el Desarrollo Social, the International Union Against Tuberculosis | Implementation activities and legal defence efforts in aid of tobacco control | Colombia |
| **129** | Villo, S., Halme, M., Ritvala, T. | Theorizing MNE-NGO conflicts in state-capitalist contexts: Insights from the Greenpeace, Gazprom and the Russian state dispute in the Arctic | 2020 | Qualitative | Extractive | 2010-2017 | Greenpeace, Bellona, WWF, Russian Bird Conservation Union | Advocated for transparency regarding Prirazlomnaya’s oil spill response plan | Arctic region |
| **130** | Viveros, H. | Unpacking stakeholder mechanisms to influence corporate social responsibility in the mining sector | 2017 | Qualitative | Extractive | 2012 | Unnamed NGOs and unions | To influence corporate social responsibility in the mining sector | Chile |
| **131** | Wapner, P. | Politics beyond the state: environmental activism and world civic politics | 1995 | Qualitative | General | 1960s – 1990s | Transnational environmental activist groups (TEAGS) | To convince actors to make decisions and act in deference to environmental awareness | Global |
| **132** | Weber, A.K., Partzsch, L. | Barking up the right tree? NGOs and corporate power for deforestation-free supply chains | 2018 | Qualitative | Extractive,  Food,  General | 2016 - 2018 | The Accountability Framework Initiative (AFi), Transparency for Sustainable Economies (Trase). | Advocacy for greater accountability and transparency | Global |
| **133** | Will, M.G., Pies, I. | Discourse Failures and the NGO Sector: How Campaigning Can Undermine Advocacy | 2017 | Qualitative | Food | 2007-2011 | Better Markets, unnamed German NGOs | Greater regulation of financial speculation with agricultural commodities | USA, Germany |
| **134** | Wong, C.K., Wan, S.H.M., Yu, I.T.S. | History of asbestos Ban in Hong Kong | 2017 | Qualitative | Asbestos | N/A | Hong Kong Workers’ Health Centre, Hong Kong Construction Industry Employees General Union, Pneumoconiosis Mutual Aid Association, Association for the Rights of Industrial Accident Victims | Advocacy for better protection and compensation for frontline industrial workers and the general public | China |
| **135** | Woronov, T. | Waging Lawfare: Law, Environment and Depoliticization in Neoliberal Australia | 2019 | Qualitative | Extractive | 2010 - 2016 | Places you Love Alliance (comprised of Mackay Conservation Group, WWF, Greenpeace, The Wilderness Society, and the Australian Conservation Foundation) | Advocated for a new generation of national environment laws. | Australia |
| **136** | Wright, B. | Non-governmental organizations and indifference as a human rights issue: the case of the Nigerian oil embargo | 2002 | Qualitative | Extractive | 1995 - 1998 | Movement for the Ogoni People, EarthAction coalition, International Roundtable on Nigeria (coalition of NGOs) | Advocacy for adherence to human rights and international environmental issues. | Ogoniland, Nigeria |
| **137** | Yadav, A., Kumar, S., Chatterjee, M., Sharma, N., Shrivastav, R., Bassi, A. | Awareness to action through multi-channel advocacy for effective tobacco control in India: A case study from Bihar | 2018 | Qualitative | Tobacco | 2009 | Socio Economic and Educational Development Society, Health Related Information Dissemination Amongst Youth. | To improve the health status of people by promoting health awareness and informing health activism. | Bihar, India |
| **138** | Yanuardi, Y., Vijge, M.J., Bierman, F. | Improving governance quality through global standard setting? Experiences from the Extractive Industries Transparency Initiative in Indonesia | 2021 | Qualitative | Extractive | 2012-2019 | Publish What You Pay Indonesia | To ensure that extractive industries enhance people’s quality of life | Indonesia |
| **139** | Yaziji, M., Doh, J.P. | NGOs and Corporations:  Conflict and Collaboration | 2009 | Qualitative | Extractive, Food | N/A | Case study 1: Environmental Defense, National Resource Defense Council  Case study 2: Global Resistance Center for Science and the Environment, NGO FORCE, WWF.  Case study 3:Global Witness, Amnesty International  Case study 4: Oxfam, Oxfam GB, Novib (Oxfam Netherlands) | Aims: preventing new coal power plants (1), seeking bans on soft drinks and government regulation (2), banning conflict diamonds (3), encouraging CSR (4). | Case 1: US.  Case 2: US and India.  Case 3: global.  Case 4: Indonesia. |
| **140** | Yeophantong, P. | China and the Accountability Politics of Hydropower Development: How Effective are Transnational Advocacy Networks in the Mekong Region? | 2020 | Qualitative | Extractive | 2010 - | International Rivers, Earth Rights International, Burma Rivers Network (BRN) and the Save the Mekong coalition, The Kachin Development Networking Group, and the Burma Environmental Working Group | Adoption of more ecologically-responsible policy and investments | Cambodia, Laos, Myanmar |
| **141** | Young, K. | Can non-governmental organisations (NGOs) and the state collaborate? A look into the emergence of NGOs and resource extraction in Greenland | 2004 | Qualitative | Extractive | 2013 -2016 | The Coalition for Better Citizen Involvement of Large-Scale Projects and Other Resource Activities | Advocacy for better public participation in Greenland | Greenland |
| **142** | Yusef, H.O., Omoteso, K. | Combating environmental irresponsibility of transnational corporations in Africa: an empirical analysis | 2016 | Qualitative | Extractive | N/A | Movement for the Survival of Ogoni People, Centre for Constitutional Rights, Earth Rights International, Vereniging Milieudefensie (Friends of the Earth Netherlands) | To advocate for public accountability of TNCs on a range of issues including environmental and human rights | Nigeria / litigation pursued USA, Netherlands, UK |
| **143** | Zajak, S | Transnational private regulation and the transformation of labour rights organizations in emerging markets: new markets for labour support work in China | 2013 | Qualitative | General | N/A | Unnamed NGOs | Aim to regulate industry practices and standards | Guangdong Province, China |
| **144** | Zhou, S. | What Difference Would a Binding International Legal Instrument on Alcohol Control Make? Lessons from the World Health Organization Framework Convention on Tobacco Control's Impact on Domestic Litigation | 2021 | Qualitative | Tobacco, Alcohol | 2003 - | Unnamed NGOs | Aim of replicating the successes of the WHO FCTC for alcohol control | Global |
